# Visualizing Oscillations in Brain Slices With Genetically Encoded Voltage Indicators

**DOI:** 10.3389/fnana.2021.741711

**Published:** 2021-11-02

**Authors:** Jun Kyu Rhee, Yayoi Iwamoto, Bradley J. Baker

**Affiliations:** ^1^Division of Bio-Medical Science and Technology, KIST School, Korea University of Science and Technology (UST), Seoul, South Korea; ^2^Brain Science Creative Research Center, Brain Science Institute, Korea Institute of Science and Technology, Seoul, South Korea; ^3^SPEC Corporation, SPACES Otemachi, Tokyo, Japan

**Keywords:** motor cortex, neural population activity, oscillations, genetically encoded voltage indicators, ArcLight, Bongwoori-Pos6, Bongwoori-R3

## Abstract

Genetically encoded voltage indicators (GEVIs) expressed pan-neuronally were able to optically resolve bicuculline induced spontaneous oscillations in brain slices of the mouse motor cortex. Three GEVIs were used that differ in their timing of response to voltage transients as well as in their voltage ranges. The duration, number of cycles, and frequency of the recorded oscillations reflected the characteristics of each GEVI used. Multiple oscillations imaged in the same slice never originated at the same location, indicating the lack of a “hot spot” for induction of the voltage changes. Comparison of pan-neuronal, Ca^2+^/calmodulin-dependent protein kinase II α restricted, and parvalbumin restricted GEVI expression revealed distinct profiles for the excitatory and inhibitory cells in the spontaneous oscillations of the motor cortex. Resolving voltage fluctuations across space, time, and cell types with GEVIs represent a powerful approach to dissecting neuronal circuit activity.

## Introduction

Historically arising from targeted surgical lesioning technique to alleviate various intractable neurological symptoms, deep brain stimulation (DBS) has developed over the years to treat Parkinson’s disease (PD), dystonia, epilepsy, cluster headache, memory loss, obsessive-compulsive disorder, Tourette syndrome (TS), as well as depression (Benabid et al., 1987, 2009; Lozano and Hamani, 2004; Bernstein and Peters, 2005). Despite the fact that DBS is almost 30 years old, how deep brain stimulation works to ameliorate such diverse neurological disorders is still unknown. While low-frequency stimulation typically causes neuronal excitation, high-frequency stimulation (HFS), a more clinically relevant DBS protocol, paradoxically causes excitation of axons in some neurons and inhibition due to neurotransmitter fatigue in some neurons at the target site (Garcia et al., 2003, 2005; Lee et al., 2003, 2004; Chang et al., 2008; Okun and Oyama, 2013).

Having an optical readout of activity from different components of neuronal circuits affected by DBS would help explain the therapeutic mechanism leading to improved clinical outcomes. Genetically encoded fluorescent sensors can label distinct cell types by limiting expression with cell-specific promoters. Unfortunately, calcium indicators are poor at reporting subthreshold activity including neuronal inhibition. Being able to monitor both activation and inhibition is critical for such ailments as PD and TS since the balance between these two types of activities has been altered.

Since voltage indicators are a direct measurement of changes in membrane potential, they could potentially detect the effects of DBS on restoring the balance between neuronal activation and inhibition. Unfortunately, genetically encoded voltage indicators (GEVIs) have a lower signal-to-noise ratio making them more difficult to use than their calcium counterparts. Despite this limitation, GEVIs continue to improve both in the size of the optical signal as well as in the speed of the response. A recent independent comparison of different GEVIs demonstrated that ArcLight gave the most consistent signal for widefield epifluorescence and 2-photon recordings *in vivo* (Bando et al., 2019; Milosevic et al., 2020). Here we test three different GEVIs, ArcLight-A242 (abbreviated ArcLight) (Jin et al., 2012; Han et al., 2014), and two ArcLight derived GEVIs developed in our lab, Bongwoori-Pos6 and Bongwoori-R3 (Lee et al., 2016). All three GEVIs contain a modified voltage-sensing domain from the *Ciona intestinalis* voltage sensing phosphatase first used in VSFP2.1 (Dimitrov et al., 2007). Bongwoori-Pos6 and Bongwoori-R3 have improved kinetics compared to ArcLight (Table 1) due to mutations in the voltage sensing domain, A154D and R229I. In addition, Bongwoori-R3 has a positively shifted V_1/2_ and is responsive to more depolarized membrane potentials than ArcLight and Bongwoori-Pos6.

**TABLE 1:** Characteristics of the GEVIs used in this study.

**ArcLight-A242**	**Bongwoori-Pos6**	**Bongwoori-R3**
V_1/2_ = −26 mV	V_1/2_ = −28 ± 3 mV	V_1/2_ = −3 ± 1 mV
Fast τ_*ON*_ = 10 ± 1 ms (65 ± 3 %) Slow τ_*ON*_ = 53 ± 4 ms	τ_*ON*_ = 6 ± 1 ms (100 %)	Fast τ_*ON*_ = 7 ± 1 ms (90 ± 1 %) Slow τ_*ON*_ = 45 ± 1 ms
Fast τ_*OFF*_ = 22 ± 1 ms (70 ± 4 %) Slow τ_*OFF*_ = 74 ± 4 ms	Fast τ_*OFF*_ = 8 ± 1 ms (97 ± 3 %) Slow τ_*OFF*_ = 80* ms	Fast τ_*OFF*_ = 6 ± 1 ms (91 ± 1 %) Slow τ_*OFF*_ = 46 ± 6 ms
%ΔF/F_0_^†^ = −19.7 ± 0.5%	%ΔF/F_0_^†^ = −10.7 ± 0.1%	%ΔF/F_0_^†^ = −15.4 ± 0.0%
SNR^†^ = 22.2 ± 1.6	SNR^†^ = 6.7 ± 1.1	SNR^†^ = 9.6 ± 0.8

*Adapted from Han et al. (2014) and Lee et al. (2016).*

*^†^Pixels correlated for action potentials (Lee et al., 2016).*

*Only one cell showed slow τ_*OFF*_ at a 100 mV pulse (Lee et al., 2016).

Disrupting the basal ganglia output in non-human primates by applying the GABA_*A*_R antagonist bicuculline to focally disinhibit the dorsal sensorimotor putamen results in isolated facial and somatic motor tics resembling those observed in the human TS patients (McCairn et al., 2009, 2016; Worbe et al., 2009, 2013), and focally disinhibiting nucleus accumbens results in vocal tics (McCairn et al., 2016). Local field potential (LFP) recordings along the cortico-basal ganglia-thalamo-cortical loop of the motor network in non-human primates as well as human patients during the occurrence of motor tics show an abrupt pronounced spike followed by oscillations phase coupled in the alpha frequency range (7–14 Hz) in the thalamus, basal ganglia, anterior cingulate cortex, as well as the primary motor cortex (McCairn et al., 2009; Marceglia et al., 2017), indicating that the acute disinhibition of GABA_*A*_R inhibitory circuit in the dorsal sensorimotor putamen generates oscillations in the thalamus and basal ganglia that propagate ipsilaterally through the anterior cingulate to the motor cortex.

A direct pharmacological disinhibition of GABA_*A*_R inhibitory network in the rat primary motor cortex *in vivo* (Castro-Alamancos, 2000), mouse frontal agranular neocortex *ex vivo* (Castro-Alamancos and Rigas, 2002), and guinea pig sensorimotor cortex *ex vivo* (Gutnick et al., 1982; Connors, 1984), results in extracellularly recorded rhythmic ∼10 Hz (7–14 Hz) large amplitude oscillations consisting of a large negative spike followed by five to fifteen low amplitude negative spikes riding atop a positive wave. Therefore, to ascertain the feasibility of eventually monitoring the DBS in the basal ganglia and the corresponding motor cortex oscillations *in vivo*, we decided to directly disinhibit the GABA_*A*_R inhibitory network in the mouse motor cortex *ex vivo* with bicuculline to test whether or not these three GEVIs could optically report neuronal activity in the motor cortex.

Unlike voltage sensitive dyes (VSDs), by using a combination of cre transgenic mice and double-floxed inverted open reading frame (DIO) expression system, GEVIs can be selectively targeted in a cell type specific manner for expression to investigate specific parts of the neuronal circuit.

In the motor cortex, CaMKIIα+ cells are the excitatory glutamatergic pyramidal cells (Jones et al., 1994). Low success rate for paired patch clamp recordings of CaMKIIα+ pyramidal cells in the mouse, rat, and cat neocortex indicate that pyramidal cells have low local interconnectivity, with connection probability ratios ranging from 1:4, 1:9, 1:10, 1:12, 1:14, 1:17, to 1:21 (Mason et al., 1991; Thomson and Deuchars, 1997; Thomson et al., 2002; Holmgren et al., 2003; Markram et al., 2015; Reimann et al., 2015; Seeman et al., 2018).

PV+ cells are a subset of GABAergic inhibitory interneurons densely connected to the nearby excitatory pyramidal cells to inhibit the somas, proximal dendrites, and axon initial segments of the pyramidal cells (Pouille and Scanziani, 2001; Packer and Yuste, 2011; Rudy et al., 2011). Paired patch clamp recordings of fast-spiking PV+ cells in the neocortex show that 61% of the pairs formed electrical synapses and 78% of the pairs formed GABAergic chemical synapses (Galarreta and Hestrin, 2002). Paired recordings of a CaMKIIα+ pyramidal cell and a fast-spiking PV+ cell reveal connection probability ranging from 75 to 50% and reciprocal connection probability ranging from 52 to 25%. Holmgren et al. (2003) estimated that of the 628 CaMKIIα+ pyramidal cells in a 200 μm × 200 μm cylindrical column of a neocortex, 492 CaMKIIα+ pyramidal cells would innervate an interneuron, 487 CaMKIIα+ pyramidal cells would receive inhibitory inputs from the same interneuron, and 432 CaMKIIα+ pyramidal cells would be reciprocally connected to the same interneuron in return. In comparison, within the same volume, one CaMKIIα+ pyramidal cell may receive synaptic inputs from only 33 other CaMKIIα+ pyramidal cells (Holmgren et al., 2003). Given such extensive connections PV+ cells make to other neurons as well as themselves in the neocortex, it is not surprising that PV+ cells participate widely in physiological and pathological oscillations (Avoli and de Curtis, 2011; Muldoon et al., 2013, 2015; Hongo et al., 2015; Serafini et al., 2016; Smith and Schevon, 2016).

Markram et al. (2015) did not estimate similar connectivity differences between excitatory and inhibitory cells; they found that there is less pyramidal cell to inhibitory interneuron connection than inhibitory interneuron to pyramidal cell connection in layer 2/3. Based on their electron miscroscopy and electrophysiology data, however, they did predict 3.6 synapses per excitatory connection and 13.9 synapses per inhibitory connection (Markram et al., 2015).

By targeting expression of a GEVI to CaMKIIα+ pyramidal cells or to PV+ inhibitory interneurons, we further explored whether or not we could derive any meaningful insight into each specific cell type’s role in generating oscillations in the motor cortex. Oscillations were also reported when the GEVI expression was restricted to CaMKIIα+ excitatory pyramidal cells or to PV+ inhibitory interneurons. The oscillation profiles of the two cell types were different, hinting at their potentially differing roles in initiation and propagation of oscillations.

In summary, we sought to visualize the activity of a neuronal circuit in the motor cortex with three GEVIs of the ArcLight family. All three GEVIs tested were able to optically report bicuculline-induced spontaneous oscillations in the mouse brain slices of the primary motor cortex *ex vivo*, and their representation of oscillations were dependent on the kinetics and the voltage range of each GEVI. The results presented here demonstrate that ArcLight family of GEVIs should be able to monitor the effects of DBS on the motor cortex.

## Materials and Methods

### Animals

C57BL/6NHsd adult mice were either purchased from Koatech (Pyeongtaek, South Korea) or from KiSAF (Korea Institute of Science and Technology, Seoul, South Korea). Ca^2+^/calmodulin-dependent protein kinase II α -Cre adult mice [abbreviated CaMKIIα-cre #005359 B6.Cg-Tg (Camk2a-cre)T29-1Stl/J, Jackson Laboratory, Bar Harbor, ME, United States] (Tsien et al., 1996) and parvalbumin -Cre adult mice (abbreviated PV-cre. #008069 B6;129P2-Pvalb^*tm1(cre)Arbr*^/J, Jackson Laboratory, Bar Harbor, ME, United States) (Hippenmeyer et al., 2005) were bred in the specific pathogen-free animal facility at KiSAF (Korea Institute of Science and Technology, Seoul, South Korea) housed under a controlled 12 h light/dark cycle and temperature, with access to food and water *ad libitum*. Experiments were performed on mice at least 10 weeks old, both male and female. All procedures were approved and supervised by the Institutional Animal Care and Use Committee of Korea Institute of Science and Technology (approval No. KIST-2014-002, KIST-2019-012 and KIST-2019-099).

**Table:** For each type of oscillation observed and analyzed in this paper, the following number of mice and slices were imaged.

**Mouse strain**	**GEVI**	**Number of mice**	**Number of slices**
C57BL/6NHsd	ArcLight	4	5
C57BL/6NHsd	Bongwoori-R3	8	11
C57BL/6NHsd	Bongwoori-Pos6	3	4
B6.Cg-Tg (Camk2a-cre)T29-1Stl/J	ArcLight	3	7
B6;129P2-Pvalbtm1(cre)Arbr/J	ArcLight	2	2

### Adeno Associated Virus Preparation

Genetically encoded voltage indicators were cloned into a pAAV plasmid containing a human synapsin promoter, the woodchuck hepatitis virus post-transcriptional regulatory element, hGH poly adenylation signal, and adeno associated virus (AAV) serotype 2 inverted terminal repeat sequences. For Ca2+/calmodulin-dependent protein kinase II α and parvalbumin specific expression, genetically encoded voltage indicators were cloned in reverse into a pAAV plasmid containing the double-floxed inverted open reading frame (DIO) cassett right after the human synapsin promoter.

AAVs were either purchased from the Upenn viral vector core, KIST virus facility, or produced in-house according to the protocol provided by the Salk Institute viral vector core facility with modifications (Kim et al., 2015). AAVpro 293T cells (#632273, Takara Bio, Shiga, Japan) were co-transfected with a mixture of three plasmids: a pAAV plasmid, the pHelper plasmid (#6672, Takara Bio, Shiga, Japan), and the pRC1 serotype 2/1 plasmid (#6672, Takara Bio, Shiga, Japan), using the calcium phosphate transfection method. 72 h after the transfection, cells were harvested and lysed by multiple rounds of freeze-thaw and tituration using a syringe and a needle, while simultaneously Benzonase nuclease (E1014, Sigma-Aldrich, Merck KGaA, Darmstadt, Germany) treated to reduce viscosity. Released AAV particles were collected *via* Iodixanol gradient ultracentrifugation at 183,000 *g* for 47 min with the NVT90 rotor (#362752, Beckman Coulter, Indianapolis, IN, United States), dialyzed in PBS with sorbitol, concentrated by centrifugal filter devices (30 KDa NMWL, Amicon Ultra-4, Merck Millipore, Cork, Ireland), and stored at −80 °C until use. Titers of the purified AAVs were estimated by performing a quantitative PCR on DNase-I treated samples, and are denoted genome content per mL (GC/mL).

### AAV Injection

Prior to injection, a glass injection pipette was pulled and fashioned from a glass capillary and then cut using a microforge to form an opening of 25–50 μm. All transcranial injections of mice between the ages of 10–15 weeks were conducted under 1.5–3% general isoflurane anesthesia. After initial induction with anesthesia, each mouse had its head affixed to the compact mouse stereotaxic frame (MCI neuroscience, East Sussex, United Kingdom). Fur over its cranium was shorn and cleaned with 70% ethanol. Commercially available hair removal cream was used to completely remove all fur to prevent the introduction of any hair into an open incision during the injection. The naked skin was then disinfected with povidone-iodine. The cranium over the right motor cortex was exposed by a midline incision. A hole through the cranium was made 0.9 mm rostral and ± 1.1–1.25 mm lateral from the bregma by a metal rotary tool kit (Foredom K.1070-2, Blackstone Industries, Bethel, CT, United States) with a drill bit size United States ¼, 0.5 mm. The glass injection pipette containing an AAV was placed over the hole and lowered so that the tip of the glass pipette would be placed 0.5–0.7 mm under the surface of the primary motor cortex. 200–400 nL of the virus solution was slowly injected using the UltraMicroPump II and Micro4 controller (World Precision Instruments, Sarasota, FL, United States). After the surgery, mice were allowed to recover on a heating pad and then returned to their home cages once they are fully awake. AAV injected mice were used for the experiments 2–5 weeks after the injection.

**Table:** Full list of AAVs utilized in this study are as follows.

**Adeno Associated Viruses**	**Titer (GC/mL)**	**Source**
AAV1.hSyn.ArcLightD. WPRE.SV40 (Addgene 36857P)	1.5 × 10^13^	UPenn vector core
AAV2/1-hSyn-ArcLight-A242	3.0 × 10^13^	Produced in-house
AAV1.hSyn.Flex. ArcLightDco.WPRE. SV40 (Addgene 36857M)	1.4 × 10^13^	UPenn vector core
AAV2/1-hSyn-DIO-ArcLight-A242	1.9 × 10^13^	Produced in-house
AAV2/1-hSyn-Bongwoori-R3	7.6 × 10^12^	KIST virus facility
AAV2/1-hSyn-Bongwoori-R3	4.0 × 10^13^	Produced in-house
AAV2/1-hSyn-Bongwoori-Pos6	8.2 × 10^12^	KIST virus facility
AAV2/1-hSyn-Bongwoori-Pos6	1.8 × 10^13^	Produced in-house

### *Ex vivo* Slice Preparation

Chemicals were obtained from Sigma-Aldrich (Merck KGaA, Darmstadt, Germany) unless otherwise specified. 300 μm thick acute coronal slices from 12–20 week old mice were prepared using a vibratome (VT-1200, Leica, Nussloch, Germany) following either sucrose artificial cerebrospinal fluid solution (aCSF) (Geiger et al., 2002; Bischofberger et al., 2006) or *N*-Methyl-D-glucamine (NMDG) aCSF based cutting method (Ting et al., 2014, 2018).

#### Sucrose aCSF Based Cutting Method

Brain is first sliced in a cold high-sucrose aCSF containing (in mM): 75 sucrose, 25 NaHCO_3_, 2.5 KCl, 0.5 CaCl_2_, 7 MgCl_2_, 1.25 NaH_2_PO_4_, and 25 D(+)-glucose, pH 7.4, oxygenated with a 95% O_2_/5% CO_2_ gas mixture. The brain slices are then transferred to aCSF containing (in mM): 124 NaCl, 2.5 KCl, 1.25 NaH_2_PO_4_, 24 NaHCO_3_, 5 HEPES, 12.5 Glucose, 2 MgSO_4_, and 2 CaCl_2_, pH 7.4, oxygenated with a 95% O_2_/5% CO_2_ gas mixture, and incubated for 30 min at 34°C, followed by recovery for 60 min at room temperature.

#### NMDG aCSF Based Cutting Method

Brain is first sliced in a cold NMDG-HEPES aCSF containing (in mM): 124 NaCl, 2.5 KCl, 1.25 NaH_2_PO_4_⋅2H_2_O, 24 NaHCO_3_, 5 HEPES, 12.5 Glucose, 2 MgSO_4_, pH 7.4, oxygenated with a 95% O_2_/5% CO_2_ gas mixture. The brain slices are then incubated for 30 min at 34°C, during which NMDG aCSF with 2 M NaCl is added multiple times to bring up the NaCl concentration in a stepwise manner. The slices are then transferred to a HEPES holding aCSF containing (in mM): 92 NaCl, 2.5 KCl, 1.25 NaH_2_PO_4_, 30 NaHCO_3_, 20 HEPES, 25 Glucose, 5 Ascorbic acid, 2 Thiourea, 3 Pyruvate, 2 MgSO_4_, and 2 CaCl_2_, pH 7.4, oxygenated with a 95% O_2_/5% CO_2_ gas mixture, and allowed to recover for 60 min at room temperature.

### Experiment Setup

A high-speed CCD camera (Neuro CCD, RedShirt Imaging, Decatur, GA, United States) connected to the upright widefield epifluorescence microscope (Slicescope, Scientifica, East Sussex, United Kingdom) in combination with a GFP filter set (GFP-3035D-OMF, Semrock, Rochester, NY, United States), LED controller (UHPLCC-01, Prizmatix, Givat-Shmuel, Israel), an ultra high power collimated LED light source (UHP-LED-460, Prizmatix, Givat-Shmuel, Israel), and a 10x water immersion objective lens (UMPlanFL N; NA = 0.3, Olympus, Tokyo, Japan) was used for all experiments. For electrophysiology, a borosilicate glass capillary (1B150F-4, WPI, Sarasota, FL, United States) pulled to 2–3 MΩ resistance using a micropipette puller (P-97, Sutter Instruments, Novato, CA, United States), filled with aCSF and inserted into the patch clamp headstage (CV-7B, Molecular Devices, San Jose, CA, United States) and the patch clamp amplifier (MultiClamp 700B, Molecular Devices, San Jose, CA, United States), connected to the digitizer (Axon Digidata 1550B, Molecular Devices, San Jose, CA, United States), and controlled by MultiClamp 700B Commander and pCLAMP 11 softwares (Molecular Devices, San Jose, CA, United States) was used for all experiments. A perfusion chamber (RC-26G, Warner Instruments, Hamden, CT, United States) with a heating platform (PH-1, Warner Instruments, Hamden, CT, United States) affixed to a stage adapter (Series 20, Warner Instruments, Hamden, CT, United States) placed on a sample plate holder (Scientifica, East Sussex, United Kingdom) was used to perfuse the slices during the experiments. To record, each brain slice was placed in the recording chamber and perfused continuously with aCSF containing (in mM): 124 NaCl, 2.5 KCl, 1.25 NaH_2_PO_4_, 24 NaHCO_3_, 5 HEPES, 12.5 Glucose, 2 MgSO_4_, and 2 CaCl_2_, pH 7.4, oxygenated with a 95% O_2_/5% CO_2_ gas mixture, at the rate of 2–3 mL/min. The recording chamber was maintained at 33°C by a temperature control unit (TC-344B, Warner Instruments, Hamden, CT, United States). For bicuculline perfused recordings, bicuculline methochloride (0131, Tocris, Bristol, United Kingdom) dissolved in aCSF at the final concentration of 20 μM was perfused over the slices.

### Widefield Epifluorescence Imaging and Local Field Potential Recording

A time-lapse widefield epifluorescence image of the primary motor cortex was recorded using a 10x objective lens, resulting in a 500 μm × 500 μm field of view, illuminated by excitation light measuring 130 mW/mm^2^ at the focal plane. LFP of the primary motor cortex was recorded by placing a glass capillary on the surface of the brain slice. For each acquisition, NeuroPlex software (RedShirt Imaging, Decatur, GA, United States) triggered the Axon Digidata 1550B digitizer *via* data acquisition board (MSTB010-06-C1C-B-C02, Microstar Laboratories, Redmond, WA, United States) to acquire the LFP as the Neuro CCD high-speed CCD camera simultaneously acquired a 10,000 frame 10 s time-lapse recording of 80 × 80 pixels at 1 kHz. 10 s LFP recording acquired at 10 kHz by the Axon Digidata 1550B digitizer was imported *via* the data acquisition board, downsampled to 2 kHz, displayed and saved onto a single file along with the simultaneously acquired 10 s time-lapse widefield epifluorescence image by the NeuroPlex software.

### Quick Analysis During a Recording Session

Ten second time-lapse widefield epifluorescence recording of the 6,400 pixels and the corresponding 10 s LFP recording was immediately processed after acquisition using the same NeuroPlex software (RedShirt Imaging, Decatur, GA, United States). The time-lapse fluorescence signals of all the pixels within the region of interest roughly encompassing the brightest area were spatially averaged. The spatially averaged time-lapse fluorescence signal was bandpass filtered between 5 and 50 Hz using the 5th order Butterworth high-pass and low-pass filters and converted to fractional fluorescence change values using appropriate reference frames, ΔF/F_*ref*_ = (F−F_*ref*_)/F_*ref*_. The corresponding LFP recording was bandpass filtered between 5 and 50 Hz using the 5th order Butterworth high-pass and low-pass filters as well. This quick on-the-spot analysis allowed us to determine the success of each 10-s recording during a 6-h recording session.

### Movies

Movies of the oscillations in MPEG format were created by taking 50–100 frames prior to the onset of an oscillation to 50–100 frames after cessation of an oscillation. Each 80 × 80 pixel frame was spatially low pass filtered using a mean filter with a 3 × 3 pixel kernel, and the maximum and minimum of the intensity range was automatically set to maximize the dynamic range of the movie without saturating any of the pixels, and pseudocolored to better visualize the changes in fluorescence intensity. All movies were slowed down 33 fold from their 1 kHz acquisition frequency to play back at 30 frames per second or 30 Hz. For some oscillations, when the decaying tail of an oscillation became difficult to distinguish from the baseline noise, the movie was cropped to better focus the attention to more prominent parts of the oscillation and to reduce the size of movies.

### Image Analysis

Further analysis was carried out offline using MATLAB scripts (MATLAB R2019a; The MathWorks, Natick, MA, United States). Of the 6,400 pixels in a frame, dark pixels that lie outside of the slice were masked. The number of pixels masked in each recording varied from 2 to 2,298, with a median of 389 pixels. The fluorescence intensity time-lapse recording of each remaining unmasked pixel was bandpass filtered between 5 and 50 Hz using the 5th order Butterworth high-pass and low-pass filters. Each filtered signal was then normalized to fractional fluorescence change signal %ΔF/F_0_ = (F−F_0_)/F_0_^∗^100 by first fitting an exponential decay curve to the baseline, and then dividing the filtered signal by the exponential decay curve, to generate an oscillation waveform for each pixel.

#### Duration, Frequency, Power Spectrum Density, the Timing of Peak Analysis

For each observed oscillation, an unmasked pixel whose oscillation waveform had the largest %ΔF/F_0_ was chosen as the reference pixel. To come up with a representative oscillation of a recording, pixels displaying oscillation waveforms highly correlated to the reference pixel were selected in such a way that addition of any of the remaining correlated pixels will significantly worsen the signal-to-noise ratio of the averaged oscillation waveform. The number of pixels chosen varied from 118 to 1,638, with a median of 696 pixels. The time at which the representative oscillation waveform gets larger than 3σ or smaller than 3σ of the Gaussian baseline noise is considered to be the starting time point and end time point of the representative oscillation waveform, respectively. Duration is the starting time point subtracted from the end time point. The Pspectrum function applied to 10 ms segments along the oscillation waveform was used to calculate the frequency along the time domain. Converting the representative time-lapse oscillation waveform to frequency based signal *via* fast Fourier transform and then squaring the values gave power spectral density of the representative oscillation waveform along the frequency domain. The time point at which the largest peak of the representative oscillation waveform occurred was considered to be the peak time point.

#### Unsupervised k-Nearest Neighbor Algorithm to Score Anomalies From the Baseline in the Recording

A PYTHON script (Python 3.9; Python Software Foundation, Beaverton, OR, United States and PyCharm 2021.1.2; JetBrains, Prague, Czechia) utilizing the numpy, opencv-contrib-python, and scikit-learn libraries was written to automate the unsupervised learning and the scoring of anomalies using the k-nearest neighbor algorithm. First, 6,400 pixels in the 80 × 80 pixel frame were segmented into 400 groups of 4 × 4 pixels, each group of 4 × 4 pixels represented by the averaged oscillation waveform of the 16 pixels. The waveforms were not further filtered or averaged throughout the k-nearest neighbor classification. For each 4 × 4 pixel group, k-nearest neighbor algorithm was first trained by saturation sampling the first 500 ms of the baseline with a 300 ms moving window. The k-nearest neighbor algorithm then scored the rest 9,500 ms of the time-lapse recording using a 300 ms moving window by calculating the distance between the sampled recording and the *k* = 1 nearest neighbor.

#### Amplitude Symmetry Analysis

Pairwise correlations of the oscillation waveform between each of the unmasked pixels and the reference pixel were calculated, and pixels were ordered and grouped according to the strength of their correlations. Group size was limited to 18% of the total number of unmasked pixels. Oscillation waveforms of pixels in each group were averaged to represent that group. Groups of the same size that are the most inversely correlated to one another were systematically paired off until there were no more groups of equal size left to pair. The amplitude symmetry coefficient, S, of the paired groups A and B is calculated:


$$\text{S}=\frac{||s\,y\,m\,m\,e\,t\,r\,i\,c\,p\,a\,r\,t||}{||s\,y\,m\,m\,e\,t\,r\,i\,c\,p\,a\,r\,t||+||a\,n\,t\,i\,s\,y\,m\,m\,e\,t\,r\,i\,c\,p\,a\,r\,t||}$$

Where,


$$S\,y\,m\,m\,e\,t\,r\,i\,c\,p\,a\,r\,t=\frac{o\,s\,c\,i\,l\,l\,a\,t\,i\,o\,n\,w\,a\,v\,e\,f\,o\,r\,m\,A-o\,s\,c\,i\,l\,l\,a\,t\,i\,o\,n\,w\,a\,v\,e\,f\,o\,r\,m\,B}{2}$$

$$A\,n\,t\,i\,s\,y\,m\,m\,e\,t\,r\,i\,c\,p\,a\,r\,t=\frac{o\,s\,c\,i\,l\,l\,a\,t\,i\,o\,n\,w\,a\,v\,e\,f\,o\,r\,m\,A+o\,s\,c\,i\,l\,l\,a\,t\,i\,o\,n\,w\,a\,v\,e\,f\,o\,r\,m\,B}{2}$$

The symmetry coefficient of 1 indicates perfect symmetry, and 0 indicates no symmetry in amplitude over time or identical amplitudes between groups A and B.

#### Spatial Origin Analysis

For each oscillation, the earliest time at which the oscillation waveform of each unmasked pixel stays larger than 3σ of the Gaussian baseline noise for at least 5–10 ms was considered to be the initiation time point of each unmasked pixel. Initiation timepoints were ordered and binned in 1 ms intervals for 1–10 ms, 5 ms intervals for 11–40 ms, 10 ms intervals for 41–80 ms, and 20 ms intervals for 81–200 ms. For each time bin containing *n* pixels, the Hopkins statistic (Hopkins and Skellam, 1954) was calculated 20 times and averaged. A Hopkins statistic, H, was calculated by randomly selecting *n* pixels from the remaining unmasked pixels without substitution and calculating:


$$\text{H}=\frac{u}{u+w}$$

Where,


$$u=\sum_{p\,i\,x\,e\,l\,s\,i\,n\,i\,t\,i\,a\,t\,i\,n\,g\,a\,n\,o\,s\,c\,i\,l\,l\,a\,t\,i\,o\,n}\bar{E\,a\,c\,h\,p\,i\,x\,e\,l-i\,t\,s\,n\,e\,a\,r\,e\,s\,t\,n\,e\,i\,g\,h\,b\,o\,r}$$

$$w=\sum_{e\,q\,u\,a\,l\,n\,u\,m\,b\,e\,r\,o\,f\,r\,a\,n\,d\,o\,m\,l\,y\,s\,e\,l\,e\,c\,t\,e\,d\,p\,i\,x\,e\,l\,s}\bar{E\,a\,c\,h\,p\,i\,x\,e\,l-i\,t\,s\,n\,e\,a\,r\,e\,s\,t\,n\,e\,i\,g\,h\,b\,o\,r}$$

The Hopkins statistic of 0.5 indicates no clustering, close to 0 indicates uniform distribution, and 1 indicates clustering of pixels.

#### To Determine if There Are Common Neural Correlates of Oscillations

10 s recordings containing more than one oscillation were chosen for this analysis. Pairwise correlation of the oscillation waveform between each unmasked pixel and the reference pixel was calculated, and pixels were ordered and grouped into four groups according to the strength of their correlations. This correlation was first performed over each of the oscillations occurring during the 10 s recording and then performed over all the oscillations.

### Statistical Test

For comparisons across three or more groups, one-way ANOVA was used. For pairwise comparisons, Tukey’s multiple comparisons test was used. For determining significance of Pearson’s correlation coefficients in Supplementary Figure 3, Student’s *t*-test was used.

## Results

### Qualitative Description of Oscillations

**FIGURE 1:**
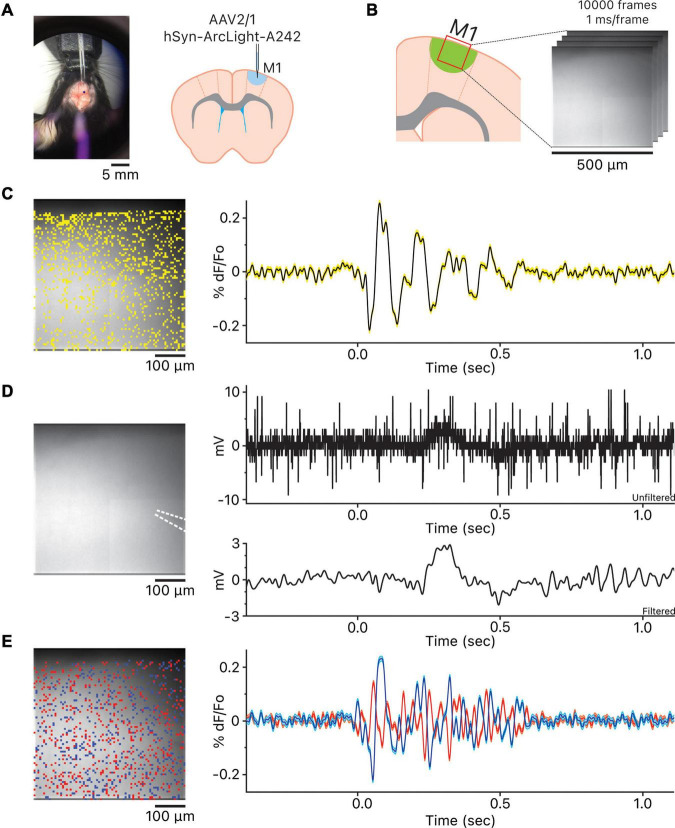
Observation of the oscillations in the motor cortex. **(A)** View of stereotaxic dissection scope over the area of AAV injection and the injection location in the primary motor cortex. **(B)** Imaging location in the primary motor cortex. **(C)** The location of the 1,007 pixels and the representative oscillation waveform produced by averaging the 1,007 oscillation waveforms. Yellow shaded area above and below the representative oscillation waveform indicates ± squared error of the mean. **(D)** The white dotted lines indicate the location of the local field potential (LFP) recording glass micropipette. To the right are the unfiltered LFP recording and the LFP recording 5–50 Hz bandpass filtered using the 5th order Butterworth high-pass and low-pass filters. Both unfiltered and filtered LFP recordings are time aligned to the representative oscillation waveform shown in panel **(C)**. **(E)** Heterogeneity of oscillation waveforms in each recording. The blue pixels are the pixels whose oscillation waveforms are the most positively correlated to the reference pixel; blue oscillation waveform is the averaged waveform, and cyan shaded area above and below the averaged waveform indicates ± squared error of the mean. The red pixels are the most negatively correlated to the reference pixel; red oscillation waveform is the averaged waveform, and pink shaded area above and below the averaged waveform indicates ± squared error of the mean. The blue and red oscillation waveforms are time aligned to both the representative oscillation waveform in C and the LFP recordings in panel **(D)**.

*Ex vivo* application of 40 μM bicuculline to block GABA_*A*_ receptors in the motor cortex slices expressing one of three GEVIs, ArcLight (Jin et al., 2012; Han et al., 2014), Bongwoori-R3, or Bongwoori-Pos6 (Lee et al., 2016) under the control of human synapsin 1 (hSyn) promoter, resulted in spontaneous widespread oscillatory activity in the upper layers of the motor cortex (Altamura et al., 2006) observable with the wide-field fluorescence microscopy (Figures 1A–C and Supplementary Movies 1–3). A stereotypical oscillation showed a large initial deflection, either depolarizing or hyperpolarizing, followed immediately by 2–19 fluctuations that typically decreased in amplitude over time (Figure 1C).

We simultaneously recorded LFPs *via* glass micropipette (Figure 1D). We did not always observe a noticeable change in potential during imaging. In some cases, LFPs were readily apparent after 5–50 Hz bandpass filtering but not for all (Figure 1D and Supplementary Figure 2). The discrepancy may have risen because we were using a glass micropipette with a single sampling point to record the LFPs instead of a multi site recording probe. If true, imaging at the tip of the glass micropipette should be more closely correlated to the LFP recording. The optical activity of the pixel at the tip of the glass micropipette reported *via* imaging (Supplementary Figure 3 and Supplementary Table 1) showed a modest but significant correlation to the LFP recording within the oscillation time window (Pearson correlation coefficient, *r* = 0.35, *p* < 0.0001, shaded gray and red boxes).

While the oscillations observed using the three GEVIs followed the stereotypical oscillatory pattern, there was considerable heterogeneity in the representative oscillation waveforms observed among all slices (Supplementary Figure 1) as well as oscillation at each pixel within a slice (Figure 1E). This heterogeneity in oscillatory activity may be the reason LFP recordings *via* a glass micropipette were less reliable for detecting an oscillation in our hands; using a multichannel linear array silicon probe may be a better solution for reliable detection of oscillations (Castro-Alamancos, 1999, 2000; Castro-Alamancos and Rigas, 2002; Castro-Alamancos and Tawara-Hirata, 2007) but that would interfere with the optical acquisition.

Unlike electrophysiological recordings, fluorescent GEVI imaging preserves the spatial information about the neural activity. Creating and comparing time-lapse fluorescent movies of the oscillations revealed that spatial neural activity similarly displayed heterogeneity. For ArcLight oscillations, eight out of 17 oscillations displayed a widespread “waving” oscillation and nine out of 17 oscillations displayed fluctuations in fluorescence in place without a perceived directionality (Supplementary Movie 1). For Bongwoori-R3 oscillations, three out of 26 oscillations displayed a widespread “waving” oscillation and 23 out of 26 oscillations displayed fluctuations in fluorescence that were punctate and in place without a perceived directionality (Supplementary Movie 2). For Bongwoori-Pos6 oscillations, two out of 20 oscillations displayed fluctuations in fluorescence that decremented in place without a perceived directionality and 18 out of 20 oscillations displayed fluctuations in fluorescence that were punctate and in place without a perceived directionality (Supplementary Movie 3).

### Comparing Oscillations Across the Three GEVIs

**FIGURE 2:**
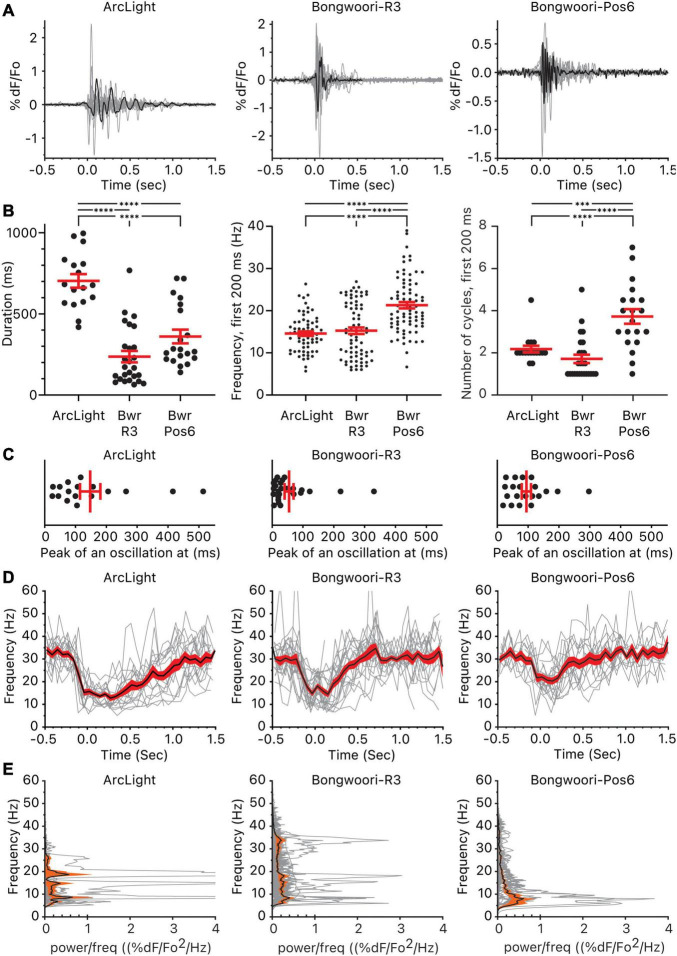
Oscillations observed using ArcLight, Bongwoori-R3, and Bongwoori-Pos6. **(A)** Individual oscillation waveform (gray) and the representative oscillation waveform (black) plotted over time. The number of mice, slices, and oscillations for each GEVI is as follows: ArcLight, 4 mice, 5 slices, 17 oscillations. Bongwoori-R3, 8 mice, 11 slices, 26 oscillations. Bongwoori-Pos6, 3 mice, 4 slices, 20 oscillations. **(B)** The total duration of the oscillations, frequency, and the number of cycles during the first 200 ms of the oscillations. ^∗∗∗^*p* < 0.001 and ^****^p < 0.0001 for one-way ANOVA and Tukey’s multiple comparisons test. **(C)** Timing of the absolute maximum %ΔF/F_0_ after the onset of the oscillations. **(D)** Frequencies of the individual oscillation waveforms (gray), the mean (black), and the squared error of the mean (red) of the frequencies plotted over time. **(E)** Power spectral densities of the individual oscillation waveforms (gray), the mean (black), and the squared error of the mean (orange) of the power spectral densities plotted over frequency. Bwr-R3, Bongwoori-R3; Bwr-Pos6, Bongwoori-Pos6.

Grouping the representative oscillations and comparing them by GEVIs, we found statistically significant differences in the duration, frequency, and the number of cycles between the three groups (Figures 2A,B). The three GEVIs differ in their voltage range. V_1/2_ depicts the voltage that corresponds to 50% of the maximal fluorescence change (Table 1). ArcLight and Bongwoori-Pos6 have V_1/2_s closer to the resting membrane potential of −70 mV than Bongwoori-R3, making them more responsive to subthreshold neuronal activity, which allows the observation of more of the decaying tails of oscillations. Indeed, durations of the oscillations differed significantly, measuring 703 ± 42 ms, 360 ± 42 ms, and 237 ± 35 ms (Mean ± SEM, *p* < 0.0001) when observed with ArcLight, Bongwoori-Pos6, and Bongwoori-R3, respectively (Figure 2B).

The ability of each GEVI to measure the duration of each oscillation does not only depend on V_1/2_. Despite having a V_1/2_ similar to Bongwoori-Pos6’s, ArcLight registered longer decaying tails of oscillations (*p* < 0.0001, Supplementary Table 1). Given that all three GEVIs share superecliptic pHluorin A227D as the fluorescent protein and are expected to have similar bleaching rates during a 10 s recording (Lee et al., 2016), differences in on/off kinetics, %ΔF/F_0_ and SNR (Table 1) play a role in each GEVI’s sensitivity to tail portions of the decaying oscillations.

One way the differing on/off kinetics of the GEVIs are reflected in the oscillations observed by each GEVI is in the detection of high-frequency components. Bongwoori-Pos6 and Bongwoori-R3 have a slightly faster τ_on_ and noticeably faster τ_off_ than ArcLight (Table 1), making them more responsive to fast voltage fluctuations (Figures 2A,E). Interestingly, for the first 200 ms of an oscillation, when the deflections are the largest in amplitude (Figure 2C), Bongwoori-Pos6 registered oscillation waveforms higher in frequency than ArcLight and Bongwoori-R3 (mean = 21.2 Hz, 14.5 Hz, 15.2 Hz. *p* < 0.0001) while Bongwoori-R3 and ArcLight registered similar frequencies (*p* = 0.7754, Supplementary Table 1) (Figures 2B,D,E). The number of oscillating cycles for the first 200 ms follows a similar trend. On average, Bongwoori-Pos6 oscillations cycled more than ArcLight and Bongwoori-R3 oscillations (mean = 3.7 cycles, 2.1 cycles, 1.7 cycles, *p* < 0.0001, one-way ANOVA, *p* = 0.0004 and *p* < 0.0001 respectively, Tukey’s multiple comparisons test) while Bongwoori-R3 and ArcLight oscillations registered similar number of cycles (*p* = 0.3980) (Figures 2A,B and Supplementary Table 1).

Bongwoori-Pos6 is more responsive to weak high-frequency voltage fluctuations and better resolved the voltage fluctuations for the first 200 ms of the oscillations than Bongwoori-R3 and ArcLight (17–26 Hz, 25–75% percentile for Bongwoori-Pos6, Figure 2B). Bongwoori-R3 with its more depolarized V_1/2_, and ArcLight with its slower off kinetics could only resolve 10–19 Hz (25–75% percentile for ArcLight and Bongwoori-R3, Figure 2B) voltage fluctuations for the first 200 ms of the oscillations.

### Using the k-Nearest Neighbor Algorithm to Detect Oscillations

We employed a k-nearest neighbor algorithm to determine how easily each GEVI can discriminate an oscillation from the baseline. K-nearest neighbor is generally used as a supervised machine learning algorithm, but for this analysis, it was employed as an unsupervised learning algorithm to calculate how oscillations are anomalous compared to the baseline (Idé, 2015). For each recording the algorithm scored, the algorithm first trained on a 500 ms stretch of the baseline. Once trained, the algorithm then scanned the rest of the recording in 300 ms segments using a moving window and scored how anomalous each segment of the recording is from the baseline by calculating the distance between each segment and its nearest neighbor in the baseline training set.

The k-nearest neighbor algorithm was able to readily distinguish ArcLight oscillations as anomalous compared to the baseline but was less capable of detecting oscillations by Bongwoori-R3 and Bongwoori-Pos6 (Supplementary Figure 4). Although ArcLight is not capable of detecting high-frequency components that Bongwoori-Pos6 can (Figures 2B,D,E), ArcLight can adequately detect the voltage fluctuations in the 10–19 Hz range for the first 200 ms of oscillations and is better at detecting the decaying tails of oscillations (Figures 2A,B and Supplementary Figure 1). These characteristics combined probably make ArcLight oscillations more amenable to the outlier detection using the k-nearest neighbor algorithm. ArcLight was therefore used to detect oscillations in distinct cell types of the motor cortex.

### Genetically Targeted Analysis of Oscillations Using Cre Recombinase tg Mice Reveals CaMKIIα+ and PV+ Cells Exhibit Different Patterns of Activity During an Oscillation

Expression of ArcLight in CaMKIIα+ cells (CaMKIIα-ArcLight) was achieved by injecting AAV1.hSyn.Flex.ArcLightDco.WPRE.SV40 into CaMKIIα-cre mice. Injection of the same AAV into PV-cre mice restricted expression to PV+ cells (PV-ArcLight) of the motor cortex. Although not all CaMKIIα+ reactive cells in the motor cortex express cre recombinase in the CaMKIIα-cre mouse, cre recombinase mediated ArcLight expression likely results in most of the pyramidal cells in layers II and III expressing ArcLight (Jones et al., 1994; Wang et al., 2013; Nakajima et al., 2021). Given that there is virtually no overlap between the CaMKIIα+ and PV+ cells (Jones et al., 1994; Wang et al., 2013), CaMKIIα+ cell and PV+ cell specific expression of ArcLight should be mutually exclusive.

**FIGURE 3:**
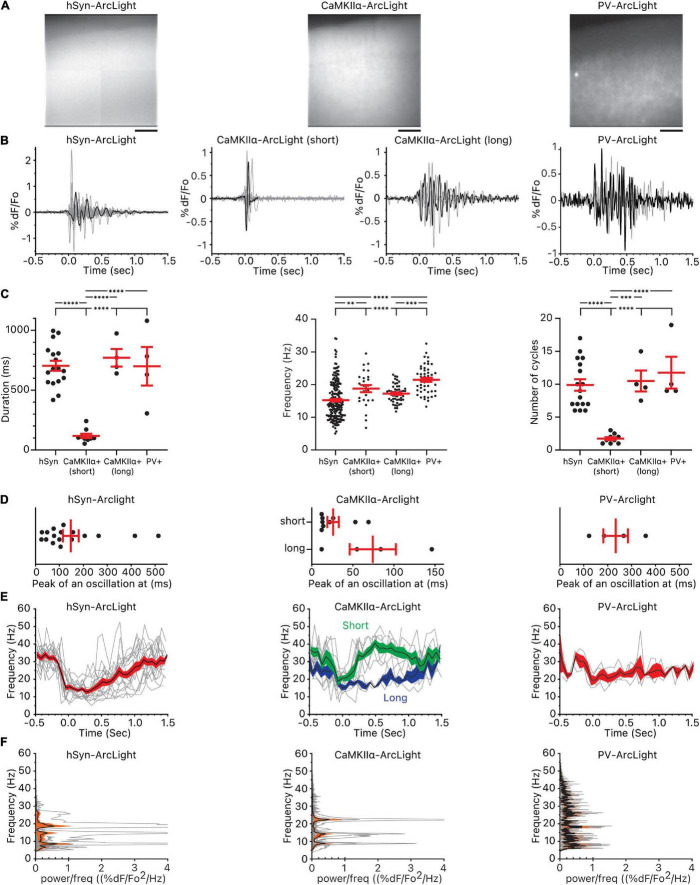
Oscillations observed in all cells, CaMKIIα+ cells, and PV+ cells. **(A)** Pattern of pan-neuronal, CaMKIIα+, and PV+ ArcLight expression in the motor cortex. Scale bar below the images are 100 μm. **(B)** Individual oscillation waveforms (gray) and the representative oscillation waveform (black) plotted over time. The number of mice, slices, and oscillations for each group are as follows: hSyn-ArcLight, 4 mice, 5 slices, 17 oscillations. CaMKIIα-ArcLight (short), 2 mouse, 5 slices, 9 oscillations. CaMKIIα-ArcLight (long), 1 mouse, 2 slices, 4 oscillations. PV-ArcLight, 2 mice, 2 slices, 4 oscillations. **(C)** Total duration of the oscillations, frequency, and the number of cycles of the oscillations. ^∗∗^*p* < 0.01, ^∗∗∗^*p* < 0.001, and ^****^p < 0.0001 for one-way ANOVA and Tukey’s multiple comparisons test. **(D)** Timing of the absolute maximum %ΔF/F_0_ after the onset of the oscillations. **(E)** Frequencies of the individual oscillation waveforms (gray), the mean (black), and the squared error of the mean (red) of the frequencies plotted over time. The squared error of the mean for CaMKIIα-ArcLight (short) is shaded in green and the squared error of the mean for CaMKIIα-ArcLight (long) is shaded in blue. **(F)** Power spectral densities of the individual oscillation waveforms (gray), the mean (black), and the squared error of the mean (orange) of the power spectral densities plotted over frequency. hSyn, hSyn-ArcLight; CaMKIIα+, CaMKIIα-ArcLight; PV+, PV-ArcLight.

Both hSyn promoter driven pan-neuronal ArcLight (hSyn-ArcLight) and CaMKIIα-ArcLight expression showed similarly uniform fluorescence; the dendritic arbors of the CaMKIIα+ pyramidal cells located in layers II and III are likely the source of ArcLight expression in layer I of CaMKIIα-ArcLight. In comparison, PV-ArcLight expresses ArcLight only in select neuropils in layers II and III (100–200 μm from the surface) in agreement with the previously published results (van Brederode et al., 1991; Lee et al., 2010; Duan et al., 2020; Figure 3A).

**FIGURE 4:**
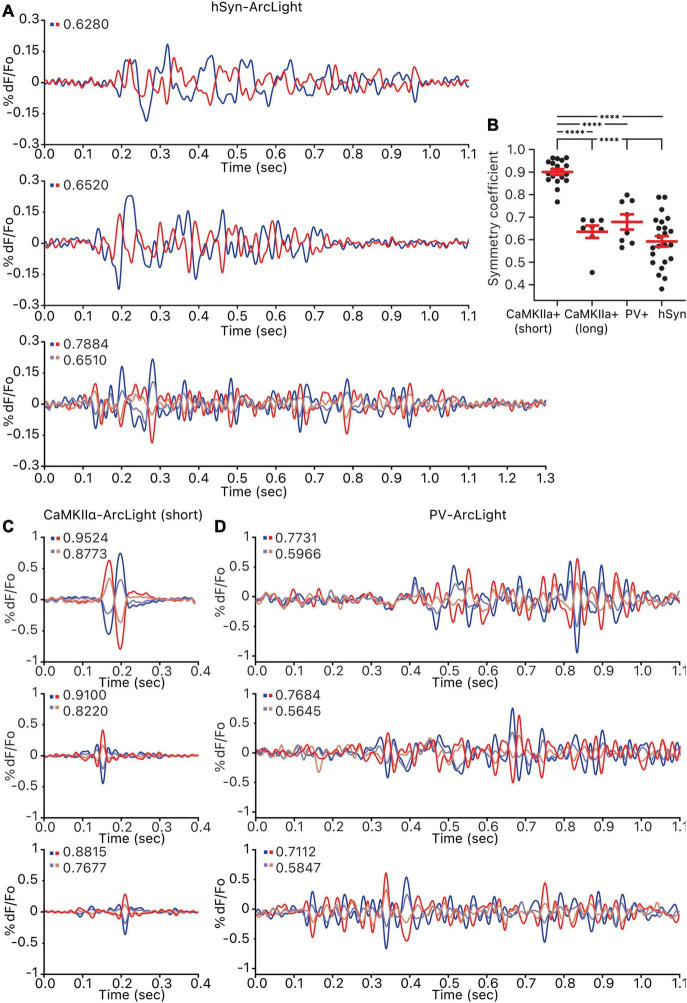
Symmetry of the oscillations observed in all cells, CaMKIIα+ cells, and PV+ cells. For each oscillation, pixels were ordered by their correlation strength to the reference pixel and grouped into 5–7 groups. Groups of the same size that are the most inversely correlated to one another were paired off, and the amplitude symmetry coefficient for each pair is calculated and shown on the top left of each axis. **(A)** Three example hSyn-ArcLight oscillations. The number of pixels in each group per pair is as follows: from the top, 1,045, 970, 1,051 and 1,051. **(B)** Amplitude symmetry coefficients of all pairs from all oscillations. ^****^p < 0.0001 for one-way ANOVA and Tukey’s multiple comparisons test. **(C)** Three example CaMKIIα-ArcLight (short) oscillations. The number of pixels in each group per pair is as follows: from the top, 945 and 945, 945 and 945, 1,018 and 1,018. **(D)** Three example PV-ArcLight oscillations. The number of pixels in each group per pair is as follows: from the top, 739 and 739, 738 and 738, 811 and 811. hSyn, hSyn-ArcLight; CaMKIIα+, CaMKIIα-ArcLight; PV+, PV-ArcLight.

CaMKIIα-ArcLight and PV-ArcLight oscillations differed in their shape, duration, number of cycles, and frequency in comparison to hSyn-ArcLight (Figures 3B,C). Unlike hSyn-ArcLight, with its large initial deflection followed by voltage fluctuations that decreased in amplitude over time, nine out of 13 CaMKIIα-ArcLight consisted entirely of short lasting 1∼2 deflections, and PV-ArcLight mostly consisted of voltage fluctuations without a discernable initial large deflection (Figure 3B and Supplementary Figure 1). From one particular CaMKIIα-ArcLight slice from one particular CaMKIIα-cre mouse, we recorded four oscillations that looked very similar in profile to hSyn-ArcLight (Figure 3B). These four CaMKIIα-ArcLight oscillations, named CaMKIIα-ArcLight (long), were similar in duration, frequency, number of cycles, the timing of their maximum absolute magnitude, and symmetry to hSyn-ArcLight oscillations (Figures 3C–E, 4B and Supplementary Table 1). The rest of the nine CaMKIIα-ArcLight oscillations consisting entirely of short lasting deflections as described above, we named them CaMKIIα-ArcLight (short).

While hSyn-ArcLight and PV-ArcLight oscillations were similar in duration (703 ± 42 ms and 700 ± 161 ms, mean ± SEM, *p* ≥ 0.9999), CaMKIIα-ArcLight (short) oscillations were shorter in duration (118 ± 19 ms) than hSyn-ArcLight and PV-ArcLight oscillations (*p* < 0.0001 and *p* < 0.0001, respectively) (Figure 3C and Supplementary Table 1). On average, hSyn-ArcLight and PV-ArcLight had about the same number of cycles during an oscillation (mean = 9.9 cycles, 11.8 cycles, *p* = 0.7274), while CaMKIIα-ArcLight (short) cycled less (mean = 1.7 cycles) than hSyn-ArcLight and PV-ArcLight (*p* < 0.0001 and *p* < 0.0001, respectively) (Figure 3C and Supplementary Table 1).

Both CaMKIIα-ArcLight (short) and PV-ArcLight displayed significantly elevated frequency than hSyn-ArcLight (mean = 18.8 and 15.2 Hz, *p* = 0.0067 and mean = 21.5 and 15.2 Hz, *p* < 0.0001, respectively). Frequency of CaMKIIα-ArcLight (short) and PV-ArcLight were similar (18.8 and 21.5 Hz, *p* = 0.1341). Interestingly, CaMKIIα-ArcLight (long) exhibited slightly lower frequency than PV-ArcLight (17.2 and 21.5 Hz, *p* = 0.0002), similar to hSyn-ArcLight (17.2 and 15.2 Hz, *p* = 0.0595) (Figures 3C–F and Supplementary Table 1).

These analyses indicate the presence of CaMKIIα+ cells in the motor cortex that oscillate briefly for 1–2 cycles at 15–22 Hz [25–75% percentiles for CaMKIIα-ArcLight (short)], and PV+ cells that oscillate longer for 9–17 cycles at 18–24 Hz (25–75% percentiles for PV-ArcLight) (Figure 3C).

Grouping the pixels of each hSyn-ArcLight, CaMKIIα-ArcLight (short), CaMKIIα-ArcLight (long), and PV-ArcLight recording by how strongly a pixel’s oscillation waveform iscorrelated to a reference pixel, and making pairwise comparisons of the most inversely correlated pixel groups exhibiting opposing oscillating waveforms, revealed that unlike hSyn-ArcLight, CaMKIIα-ArcLight (long), and PV-ArcLight oscillation waveforms, CaMKIIα-ArcLight (short) oscillation waveform displayed strong symmetry between the inversely correlated pixel groups (mean amplitude symmetry coefficient, *S* = 0.5920, 0.6352, and 0.6785 vs. 0.9004, *p* < 0.0001 for one-way ANOVA and Tukey’s multiple comparisons test) (Figure 4 and Supplementary Table 1). Neither PV-ArcLight nor CaMKIIα-ArcLight (long) oscillation waveforms displayed significantly more symmetry than hSyn-ArcLight (*S* = 0.6785 and 0.6352 vs. 0.5920, *p* = 0.6438 and 0.0984, respectively) (Figure 4 and Supplementary Table 1).

It is possible that the degree of symmetry observed in the oscillation waveforms is indicative of the cell type heterogeneity defined in each case. CaMKIIα-cre mediated expression is strictly restricted to pyramidal cells, while PV-cre mediated expression is more broadly restricted to a heterogeneous group of PV+ inhibitory interneurons. The more strictly defined CaMKIIα-ArcLight (short) oscillation waveforms are the most symmetric, while the more heterogeneous PV-ArcLight and the most heterogeneous hSyn-ArcLight oscillation waveforms are less symmetric.

The observation of CaMKIIα-ArcLight (long) oscillations similar to hSyn-ArcLight raises the possibility that CaMKIIα+ cells in the motor cortex are capable of displaying a prototypical hSyn-ArcLight-like oscillation themselves, albeit less frequently than briefly oscillating CaMKIIα-ArcLight (short). It may be that CaMKIIα-ArcLight (short) are indicative of an oscillation that initialized but failed to propagate throughout the motor cortex to result in a full fledged CaMKIIα-ArcLight (long) oscillation. Low intraconnectivity among CaMKIIα+ cells in contrast to high intraconnectivity among PV+ cells and high PV+/CaMKIIα+ interconnectivity may be a possible explanation for why oscillations initiated at the CaMKIIα+ cells would fail to propagate. Selectively expressing ArcLight only in CaMKIIα+ cells may have enabled observation of these short lasting CaMKIIα-ArcLight (short) oscillations that would normally have been buried under the background fluorescence when ArcLight is expressed panneuronally, and that may be why we did not observe short lasting CaMKIIα-ArcLight (short)-like oscillations in our hSyn-ArcLight recordings.

### No Spatial Origin of Oscillations

The advantage of optically measuring the population activity of neurons is the ability to spatially analyze the time-lapse images that can potentially guide the experimenter to a useful hypothesis for future investigation. Given the considerable heterogeneity of each pixel’s oscillation waveform (Figure 1D) and the fact that such spontaneous synchronized discharges can occur even in isolated blocks of the neocortex, mechanisms for initiating and coordinating this activity must reside within the cortex itself.

**FIGURE 5:**
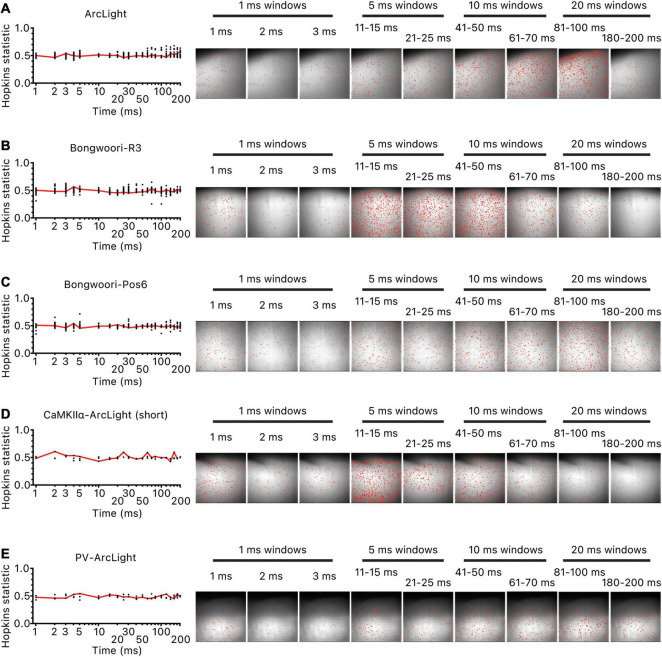
Lack of clustered origins of oscillations. For each oscillation, the initiation time point of each unmasked pixel was ordered and binned in 1/5/10/20 ms time bins for the first 200 ms of the oscillation. For each time bin, as many pixels were selected at random as the pixels in the time bin, and the Hopkins statistic was calculated. The process was repeated 20 times and averaged. The red line is the averaged values. Hopkins statistic close to 0 indicates uniform distribution, 0.5 indicates no clustering, and 1 indicates clustering of pixels. Hopkins statistic and location of pixels that started oscillating in each time bin for **(A)** representative hSyn-ArcLight oscillation, **(B)** representative hSyn-Bongwoori-R3 oscillation, **(C)** representative hSyn-Bongwoori-Pos6 oscillation, **(D)** representative CaMKIIα-ArcLight (short) oscillation, **(E)** representative PV-ArcLight oscillation.

To locate potential origins for these oscillations, we calculated the earliest time at which the magnitude of each pixel’s oscillation waveform significantly increased over the baseline noise and binned the pixels in 1, 5, 10, or 20 ms time bins for the first 200 ms of the oscillation (most oscillations peaked within the first 200 ms of the oscillation for all three GEVIs (Figure 2C). Hopkins statistics for all time bins for all the recordings were near 0.5, indicating a lack of a clear clustered spatial origin of oscillations (Figure 5 left). Visual inspection of the location of the pixels in each time bin reaffirmed the lack of an identifiable cluster of origin (Figure 5 right).

### No Shared Common Neural Substrate

Given that there are no clear spatially clustered origins of oscillations, we wanted to see if there was an indication of a spatially fixed neural substrate for these oscillations. 19 recordings (4 ArcLight, 10 Bongwoori-R3, 5 Bongwoori-Pos6) with 2 or more oscillations were analyzed to see if pixels closely correlated during one oscillation remained closely correlated for another oscillation within the same recording. Evidence of closely correlated pixels would suggest an underlying shared common neural substrate for these oscillations.

**FIGURE 6:**
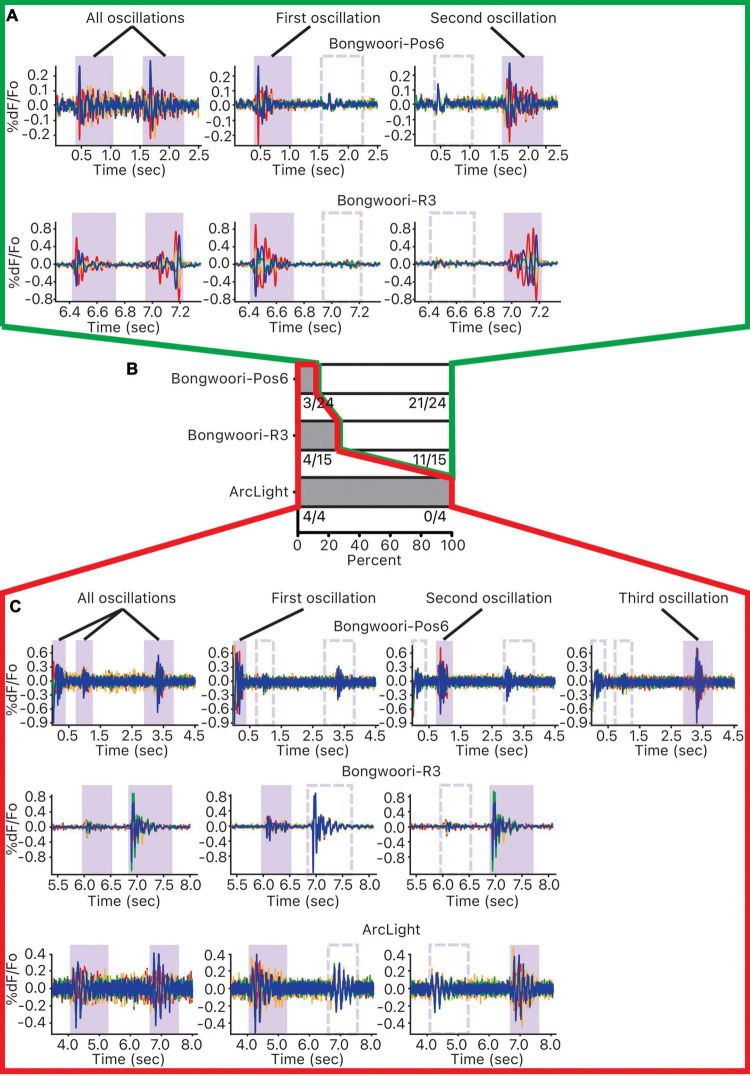
Investigating common neural substrates of oscillations. For each 10 s recording containing more than one oscillation, pairwise correlations of each unmasked pixel and the reference pixel were calculated. Pixels were ordered and grouped into four groups according to the strength of their correlations. This correlation was first performed over all the oscillations, and then each of the oscillations occurring during the 10 s recording. **(A)** Pixels that were correlated for one oscillation but not the other indicate that the pixels that oscillated together for one oscillation did not oscillate together for the other one. **(B)** For each GEVI, distribution of oscillation pairs that mostly shared oscillating pixels (gray, outlined in red) and oscillation pairs that mostly did not share oscillating pixels (white, outlined in green). **(C)** Pixels that were correlated for one oscillation as well as for the other indicate that the pixels that oscillated together for one oscillation oscillated together for the other one.

Of the 43 pairs of oscillations analyzed, 32 pairs did not share a common neural substrate, and 11 pairs did (Figure 6B). The breakdown of the result by GEVIs paints a more nuanced picture. Of the four pairs of ArcLight oscillations studied, all four pairs showed some shared common neural substrate (Figures 6B,C). But of the 15 pairs of Bongwoori-R3 and 24 pairs of Bongwoori-Pos6 oscillations studied, 11 pairs (73.3%) and 21 pairs (87.5%), respectively, showed no shared substrate (Figures 6A,B).

Because this analysis relies on correlating waveforms cycling between 10–40 Hz (Figure 2F), ArcLight oscillations with slower off kinetics may be more prone to overrepresenting averaged waveforms of each group of pixels as correlated.

With this caveat, given that the faster off kinetics and the V_1/2_s of Bongwoori-Pos6 and Bongwoori-R3 allow Bongwoori-Pos6 to better reflect the high frequency component of the oscillations in the first 200 ms, and Bongwoori-R3 to more faithfully reflect the large initial deflections of the oscillations, it may be that the initial large deflections and their high frequency components mostly do not share common substrates.

## Discussion

Oscillations of neural networks can be studied using electrodes to collect local field potentials (LFPs) (Roopun et al., 2006; Serrano-Reyes et al., 2020). Summing up the differences between a reference potential and a potential generated by all active cellular processes within a volume of brain tissue gives rise to a measurable electric field potential at the location of an extracellular electrode reflecting the neuronal population activity. Monitoring such neuronal population activity is also well suited for optical exploration using fluorescent sensors.

Advances in imaging technologies that brought us cameras of high spatial and temporal resolution have made imaging neural population activity with fluorescent probes accessible. Voltage sensitive dyes (VSDs) have been used in mice to image neural activity in the barrel cortex (Davis et al., 2011; Tsytsarev et al., 2017; Margalit and Slovin, 2018), frontal cortex (Nagasaka et al., 2017), insular cortex (Fujita et al., 2017), sensory cortex (Gollnick et al., 2016), sensorimotor cortex (Luhmann, 2017; Sreenivasan et al., 2017; Kunori and Takashima, 2019; Tang et al., 2020), and the motor cortex (Kunori et al., 2014; Kunori and Takashima, 2016) *in vivo*. In case of the motor cortex, the researchers imaged the neural activity of the M1 and M2 motor cortex 200–300 μm below the cortical surface using the VSD and were able to observe spread of neural activity from M2 to M1 following forelimb-related sensory-evoked M2 activity.

Genetically encoded voltage indicators (GEVIs) have also been utilized to image sensory cortex (Borden et al., 2017) and layer 2/3 pyramidal neurons in the whole brain (Shimaoka et al., 2017) *in vivo*, but have yet to see widespread adaptation comparable to calcium imaging or even VSDs. Voltage imaging is more difficult given that the functionally active probe is limited to the plasma membrane and that the change in membrane potential can be small. The complex morphology of many neurons further complicates matters, as the fluorescence from neuronal processes can dominate the image making it difficult to identify the individual cells exhibiting voltage transients (Nakajima and Baker, 2018). As a result of these and other challenges, there is currently no perfect GEVI capable of meeting every experimenter’s demands. The rhodopsin-based probes exhibit very fast response times but require intense illumination powers ranging from 1.6 W/mm^2^ (Piatkevich et al., 2019) to 30 mW/cell (Adam et al., 2019). The recently reported SomArchon that limits fluorescence primarily to the soma recorded an average increase in brain temperature of 1.88 ± 0.8°C during 12 s of illumination (Piatkevich et al., 2019). While such an increase may not result in tissue damage, it can definitely affect the functioning of a neuron (Owen et al., 2019).

Despite these challenges, all three GEVIs tested were able to optically report neuronal circuit activity from a population of cells in the brain slice of the motor cortex *ex vivo*. We imaged the motor cortex with ArcLight, Bongwoori-R3, and Bongwoori-pos6, three GEVIs differing in kinetics and voltage sensitivities and visualized both the neuronal excitation and inhibition during bath application of bicuculline. We were able to detect spontaneous ensemble firing of the neurons resulting in an oscillation in the motor cortex using wide-field epifluorescence microscopy. However, there were differences due to the physical characteristics of the different GEVIs tested. Oscillations observed using Bongwoori-R3 and Bongwoori-Pos6 were shorter than those observed with ArcLight, and ArcLight better registered the decaying tails of oscillations. For the first 200 ms of an oscillation, when the deflections are the largest in amplitude, Bongwoori-Pos6 oscillation waveforms registered higher frequency as well as larger number of oscillating cycles than ArcLight and Bongwoori-R3, presumably because Bongwoori-Pos6 with its V_1/2_ closer to the resting membrane potential than Bongwoori-R3 and kinetics faster than ArcLight is more responsive to weak high frequency fluctuations in the large deflections.

Comparing the oscillations observed with each of the three GEVIs demonstrate how the on/off kinetics, voltage range, and signal size of a GEVI influences the optical output of detectable neuronal activities. For instance, taking advantage of preserved spatial information about the neural activity, we were able to conclude that there were no spatially clustered origins for all oscillations regardless of the GEVI used (Figure 5). For all other analyses, however, choice of the GEVI influenced what conclusions we were able to draw. Quantitative traits such as duration, frequency, number of cycles of oscillations (Figure 2), and the result of waveform correlation analysis to determine if there is a shared common neural substrate among oscillations occurring back to back within a single recording (Figure 6) were influenced by the GEVI used to record the oscillations.

Unlike VSDs, GEVIs allow an experimenter to restrict voltage sensor expression to specific cell types using transgenic cre-recombinase mouse lines. This genetically targeted approach allows investigators to optically interrogate a desired neural circuit at both the population and a single/paired neuron level (Luo et al., 2016). Genetically targeting GEVI expression to compare pan-neuronal, CaMKIIα+, and PV+ cells revealed distinct profiles for the excitatory and inhibitory cells in the oscillations of the motor cortex. In CaMKIIα+ cells, we frequently observed brief 1–2 cycle oscillations reminiscent of initial large deflection seen in pan-neuronal oscillations. We hypothesize these may be oscillations that initialized but failed to propagate throughout the motor cortex, indicating that the CaMKIIα+ pyramidal cells may be uniquely involved in initialization of spontaneous oscillations in the motor cortex. In PV+ cells, we mainly observed long lasting voltage fluctuations without a discernable initial large deflection. We hypothesize that PV+ cells with their extensive intraconnectivity as well as interconnectivity with pyramidal cells may be uniquely involved in propagating oscillations once they are initiated. The results highlight one of the ways GEVIs can be used to generate testable hypothesis about the role of different cell types in a specific neuronal circuit activity under study.

The analyses presented here of voltage fluctuations resolved in space, time, and cell types using GEVIs present a powerful approach to dissecting the activities of neuronal circuits. Clearly, faster and brighter GEVIs with larger dynamic ranges will improve our understanding of neuronal circuits. ArcLight-based GEVIs were able to report network activity even with expression limited to distinct cell types in the motor cortex *ex vivo*, setting a foundation for monitoring the effects of DBS *in vivo*.

## Data Availability Statement

The original contributions presented in the study are included in the article/Supplementary Material, further inquiries can be directed to the corresponding author/s.

## Ethics Statement

All procedures were approved and supervised by the Institutional Animal Care and Use Committee of the Korea Institute of Science and Technology (Approval Nos. KIST-2014-002, KIST-2019-012, and KIST-2019-099).

## Author Contributions

BB and JKR planned the experiment. JKR carried out the experiments. BB, YI, and JKR analyzed the data. All authors wrote and edited the manuscript.

## Conflict of Interest

YI was employed by SPEC Corporation. The remaining authors declare that the research was conducted in the absence of any commercial or financial relationships that could be construed as a potential conflict of interest.

## Publisher’s Note

All claims expressed in this article are solely those of the authors and do not necessarily represent those of their affiliated organizations, or those of the publisher, the editors and the reviewers. Any product that may be evaluated in this article, or claim that may be made by its manufacturer, is not guaranteed or endorsed by the publisher.
