# Supplementary material for: Visualizing Oscillations in Brain Slices With Genetically Encoded Voltage Indicators
Source: Front Neuroanat. 2021 Nov 2;15:741711. doi: 10.3389/fnana.2021.741711 (PMC8592998; doi:10.3389/fnana.2021.741711)
Supplement: Supplementary file 3 [file Presentation_3.pptx]

## Slide 1
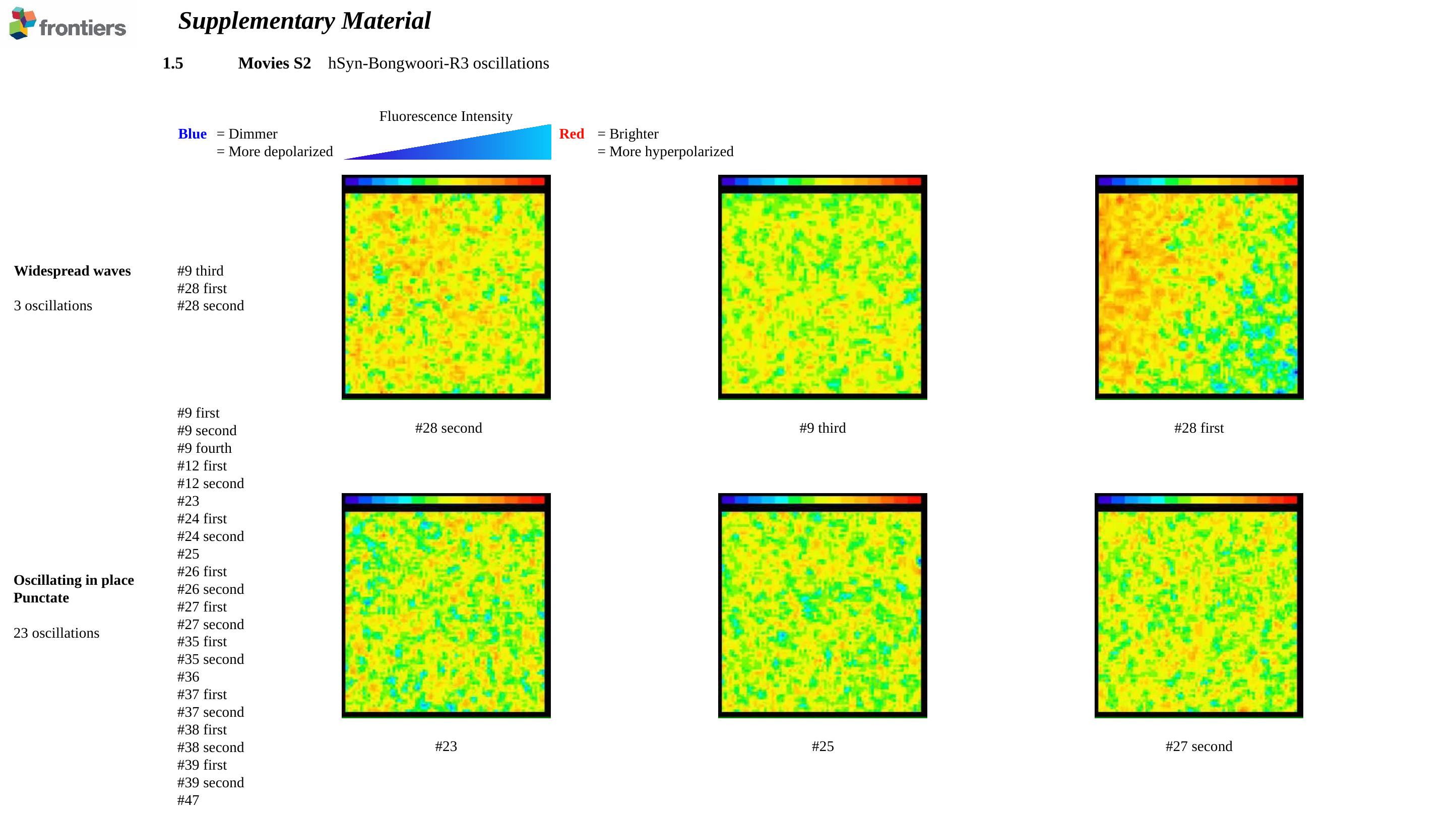

Supplementary Material
1.5	Movies S2 hSyn-Bongwoori-R3 oscillations
Fluorescence Intensity
Blue	= Dimmer
	= More depolarized
Red	= Brighter
	= More hyperpolarized
Widespread waves
3 oscillations
#9 third
#28 first
#28 second
#9 first
#9 second
#9 fourth
#12 first
#12 second
#23
#24 first
#24 second
#25
#26 first
#26 second
#27 first
#27 second
#35 first
#35 second
#36
#37 first
#37 second
#38 first
#38 second
#39 first
#39 second
#47
#28 first
#28 second
#9 third
Oscillating in place
Punctate
23 oscillations
#23
#27 second
#25

## Slide 2
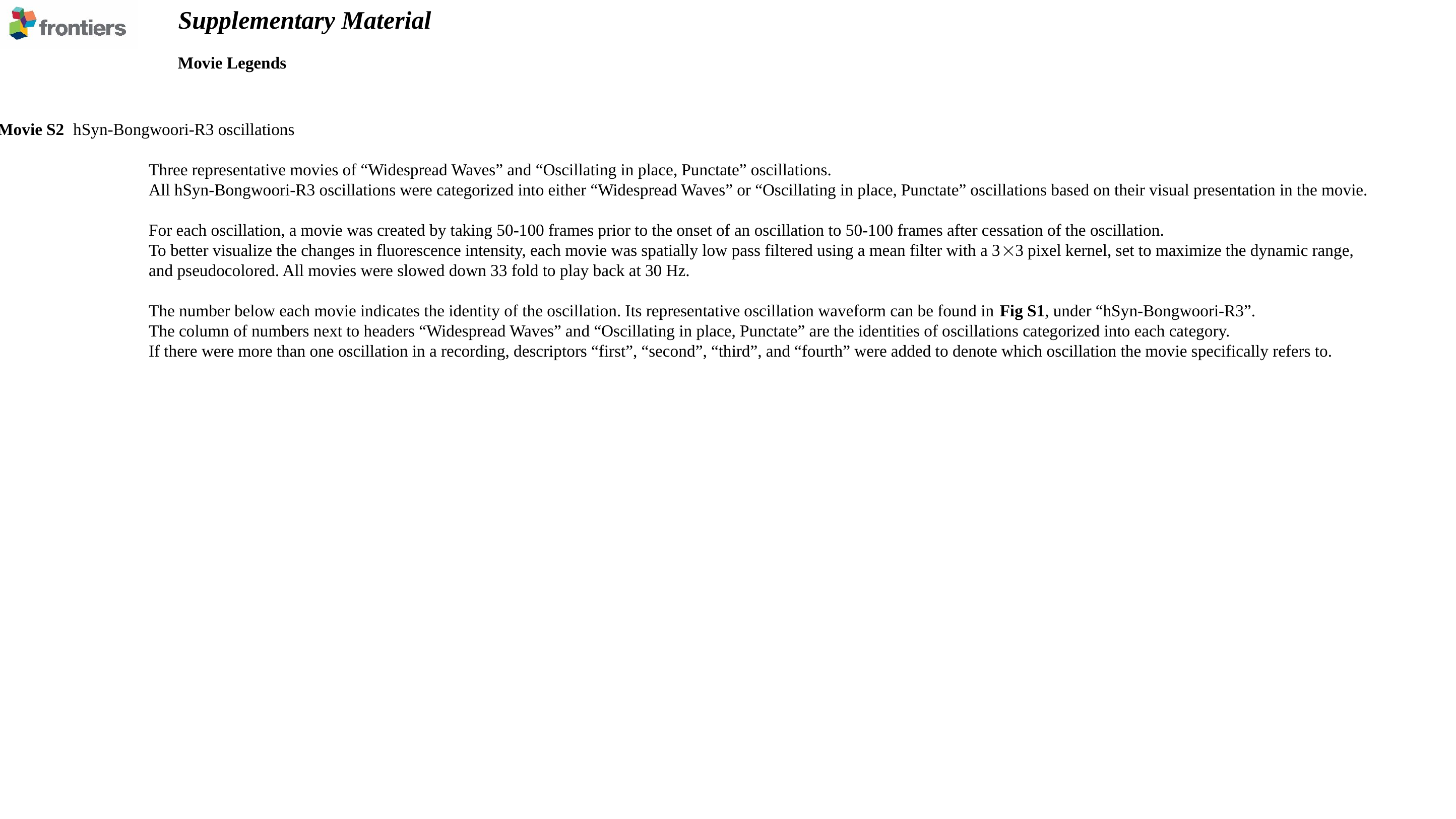

Supplementary Material
Movie Legends
Movie S2	hSyn-Bongwoori-R3 oscillations
		Three representative movies of “Widespread Waves” and “Oscillating in place, Punctate” oscillations.
		All hSyn-Bongwoori-R3 oscillations were categorized into either “Widespread Waves” or “Oscillating in place, Punctate” oscillations based on their visual presentation in the movie.
		For each oscillation, a movie was created by taking 50-100 frames prior to the onset of an oscillation to 50-100 frames after cessation of the oscillation.
		To better visualize the changes in fluorescence intensity, each movie was spatially low pass filtered using a mean filter with a 3×3 pixel kernel, set to maximize the dynamic range,
		and pseudocolored. All movies were slowed down 33 fold to play back at 30 Hz.
		The number below each movie indicates the identity of the oscillation. Its representative oscillation waveform can be found in Fig S1, under “hSyn-Bongwoori-R3”.
		The column of numbers next to headers “Widespread Waves” and “Oscillating in place, Punctate” are the identities of oscillations categorized into each category.
		If there were more than one oscillation in a recording, descriptors “first”, “second”, “third”, and “fourth” were added to denote which oscillation the movie specifically refers to.
