# Supplementary material for: Visualizing Oscillations in Brain Slices With Genetically Encoded Voltage Indicators
Source: Front Neuroanat. 2021 Nov 2;15:741711. doi: 10.3389/fnana.2021.741711 (PMC8592998; doi:10.3389/fnana.2021.741711)
Supplement: Supplementary file 4 [file Presentation_4.pptx]

## Slide 1
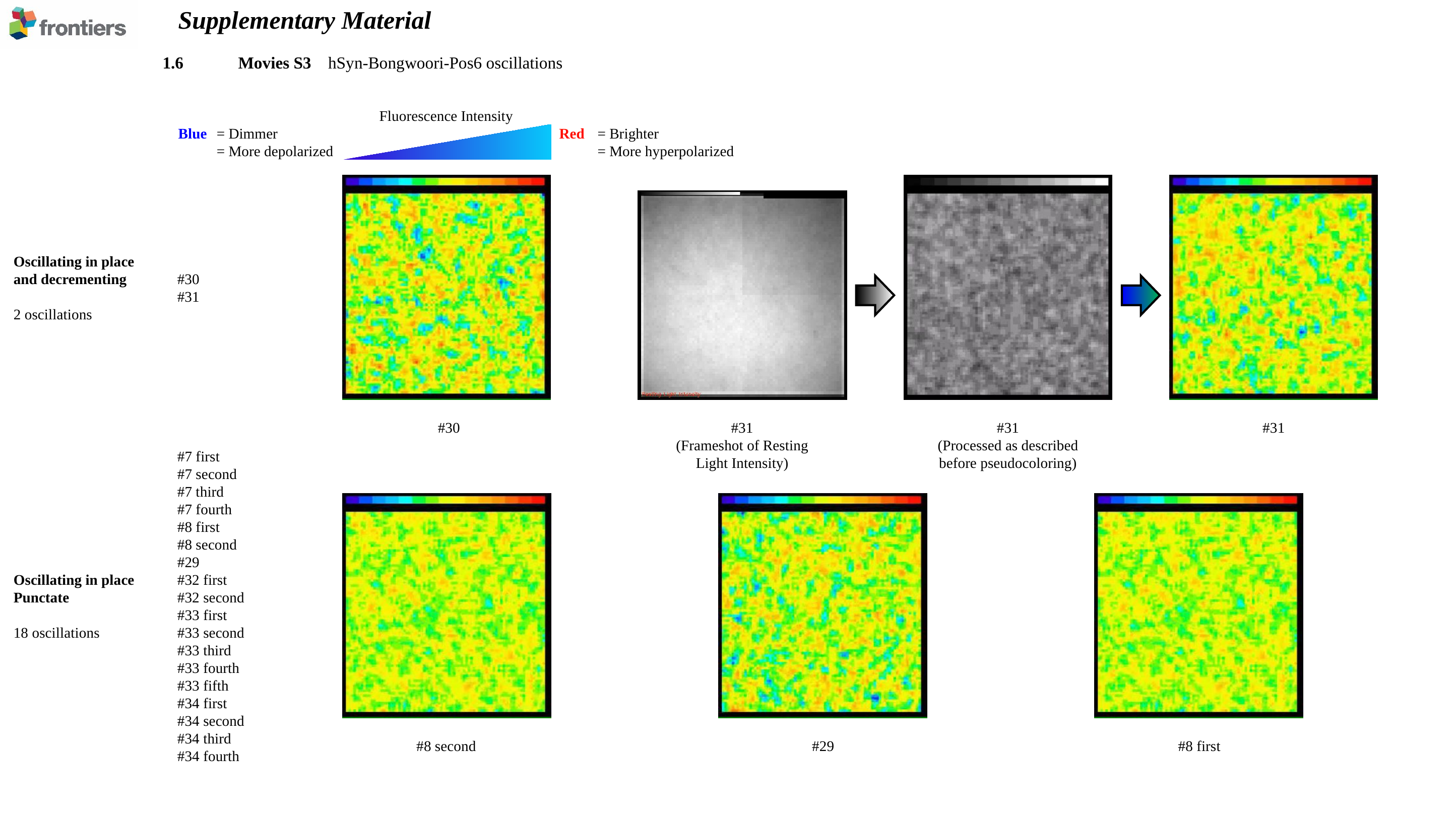

Supplementary Material
1.6	Movies S3 hSyn-Bongwoori-Pos6 oscillations
Fluorescence Intensity
Blue	= Dimmer
	= More depolarized
Red	= Brighter
	= More hyperpolarized
Oscillating in place
and decrementing
2 oscillations
#30
#31
#31
(Frameshot of Resting
Light Intensity)
#31
(Processed as described
before pseudocoloring)
#31
#30
#7 first
#7 second
#7 third
#7 fourth
#8 first
#8 second
#29
#32 first
#32 second
#33 first
#33 second
#33 third
#33 fourth
#33 fifth
#34 first
#34 second
#34 third
#34 fourth
Oscillating in place
Punctate
18 oscillations
#8 second
#8 first
#29

## Slide 2
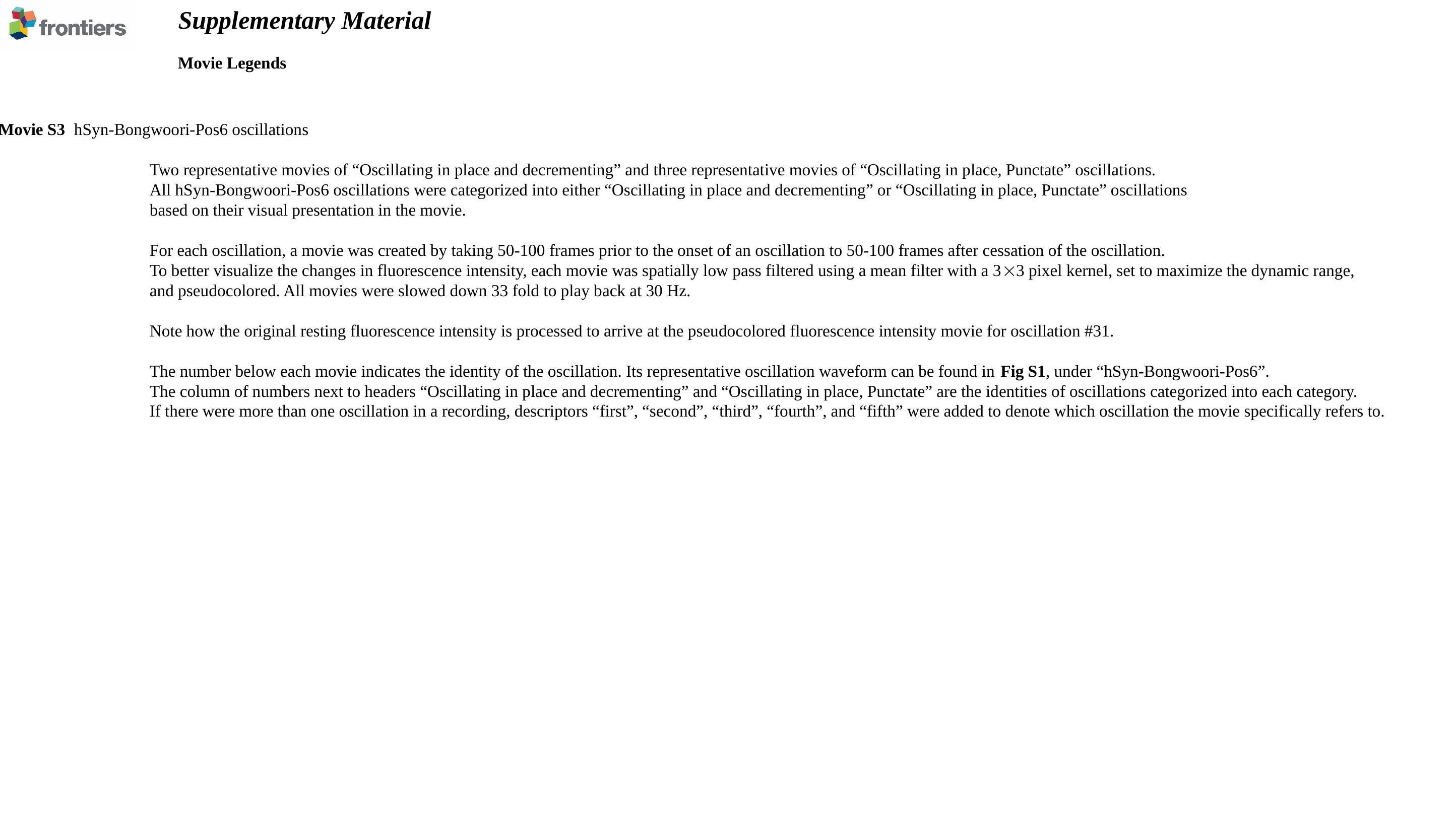

Supplementary Material
Movie Legends
Movie S3	hSyn-Bongwoori-Pos6 oscillations
		Two representative movies of “Oscillating in place and decrementing” and three representative movies of “Oscillating in place, Punctate” oscillations.
		All hSyn-Bongwoori-Pos6 oscillations were categorized into either “Oscillating in place and decrementing” or “Oscillating in place, Punctate” oscillations
		based on their visual presentation in the movie.
		For each oscillation, a movie was created by taking 50-100 frames prior to the onset of an oscillation to 50-100 frames after cessation of the oscillation.
		To better visualize the changes in fluorescence intensity, each movie was spatially low pass filtered using a mean filter with a 3×3 pixel kernel, set to maximize the dynamic range,
		and pseudocolored. All movies were slowed down 33 fold to play back at 30 Hz.
		Note how the original resting fluorescence intensity is processed to arrive at the pseudocolored fluorescence intensity movie for oscillation #31.
		The number below each movie indicates the identity of the oscillation. Its representative oscillation waveform can be found in Fig S1, under “hSyn-Bongwoori-Pos6”.
		The column of numbers next to headers “Oscillating in place and decrementing” and “Oscillating in place, Punctate” are the identities of oscillations categorized into each category.
		If there were more than one oscillation in a recording, descriptors “first”, “second”, “third”, “fourth”, and “fifth” were added to denote which oscillation the movie specifically refers to.
