# Supplementary material for: Visualizing Oscillations in Brain Slices With Genetically Encoded Voltage Indicators
Source: Front Neuroanat. 2021 Nov 2;15:741711. doi: 10.3389/fnana.2021.741711 (PMC8592998; doi:10.3389/fnana.2021.741711)
Supplement: Supplementary file 5 [file Data_Sheet_1.docx]

Supplementary Material

## Figure S1

**Fig S1.** Representative oscillation waveform of all oscillations analyzed in this article.








## Figure S2

**Fig S2.** Examples of representative oscillation waveforms and their simultaneously acquired unfiltered and filtered local field potential (LFP) recordings.





## Figure S3

**Fig S3.** Distance dependent dissociation of the LFP recording with the GEVI imaging.


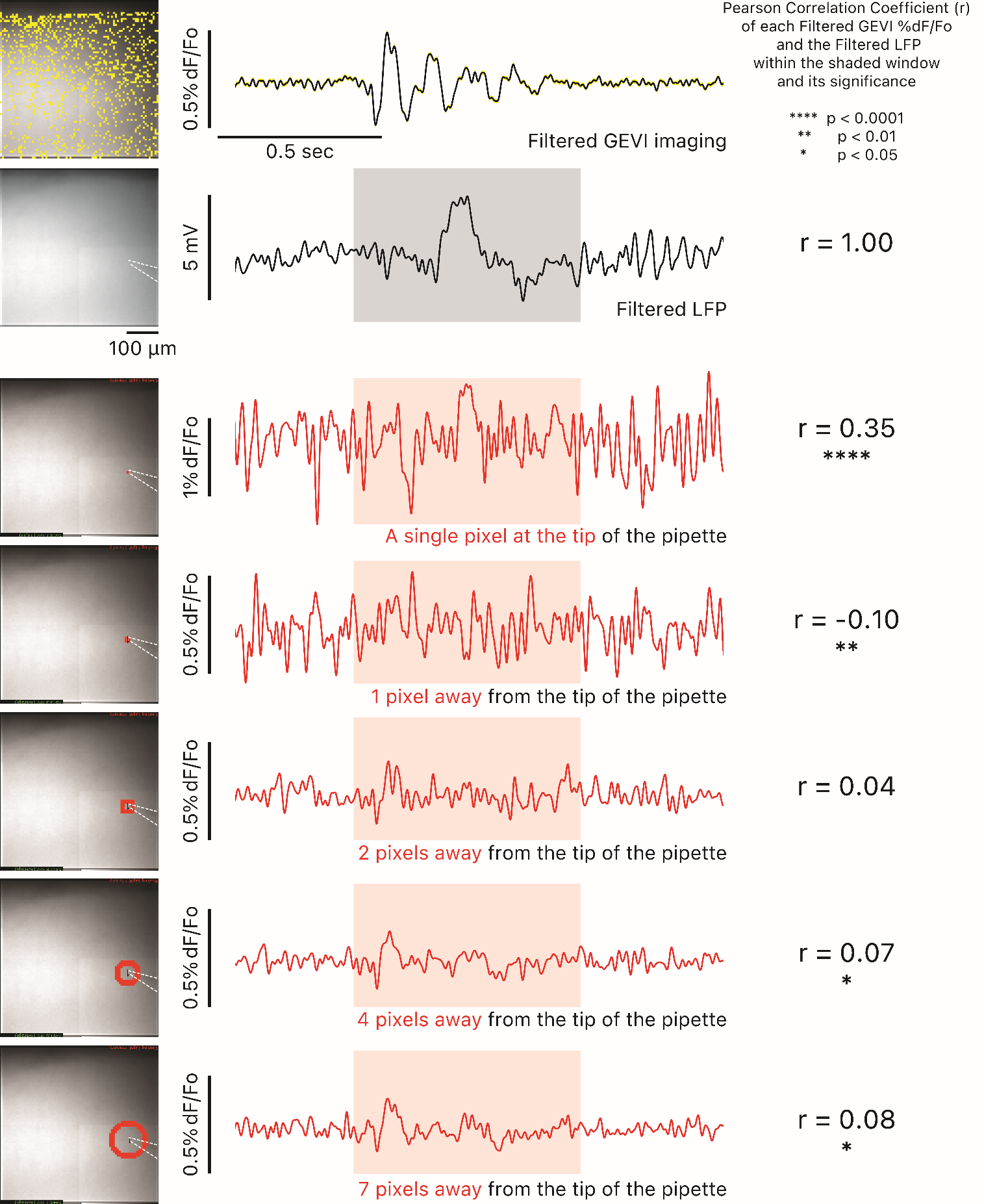


## Figure S4

##
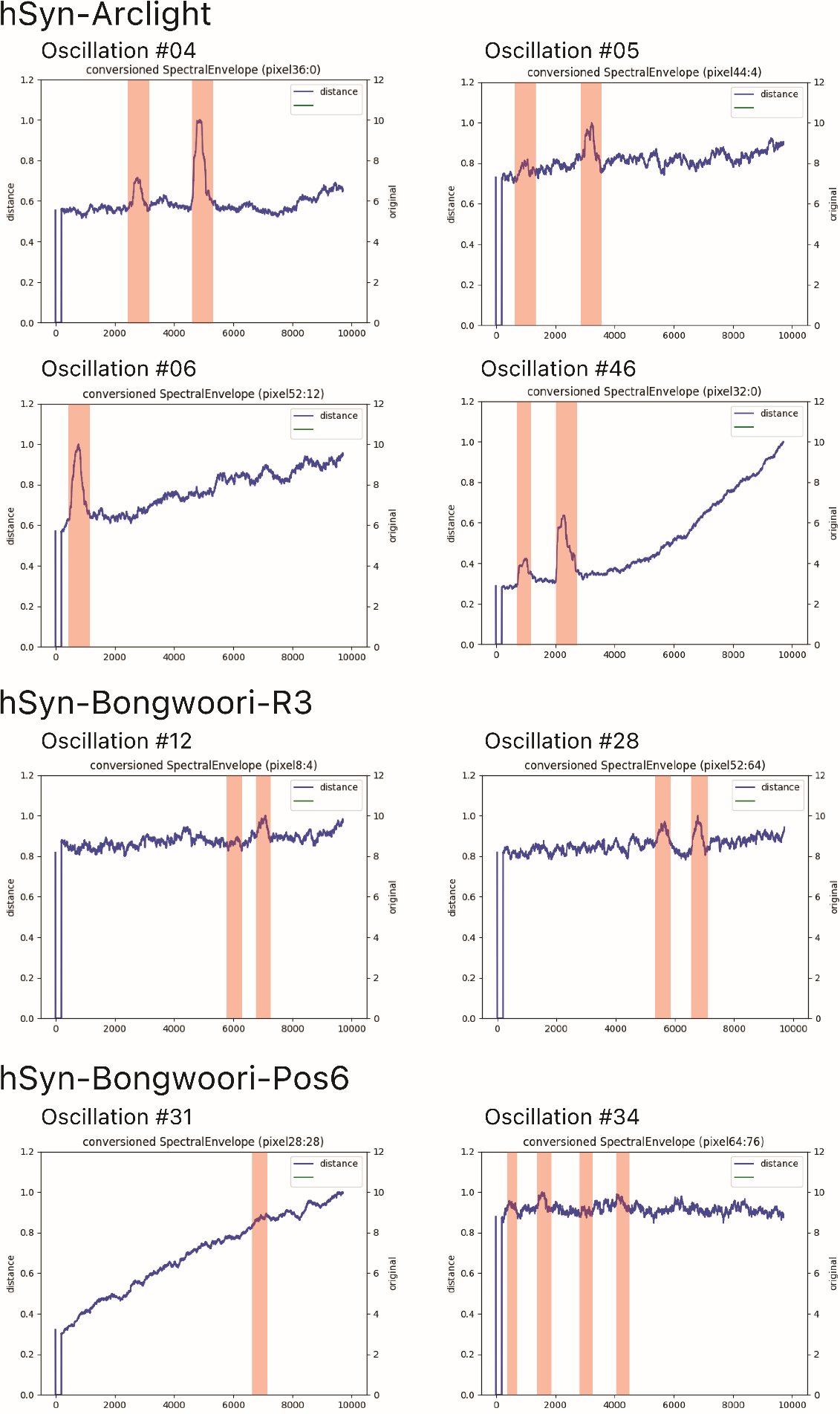
Fig S4. Representative k-nearest neighbor algorithm results for the three GEVIs.

## Table S1

**Table S1.** *P* values and statistical tests.

| **Figure** | **Groups tested** | ***p* value** | **Significance^†^** | **Statistical test** |
| --- | --- | --- | --- | --- |
| **2B**  **Duration** | ArcLight  Bongwoori-R3  Bongwoori-Pos6 | < 0.0001 | **** | One-way ANOVA |
|  | ArcLight  Bongwoori-R3 | < 0.0001 | **** | Tukey’s multiple comparisons test |
|  | ArcLight  Bongwoori-Pos6 | < 0.0001 | **** | Tukey’s multiple comparisons test |
|  | Bongwoori-R3  Bongwoori-Pos6 | 0.0622 | ns | Tukey’s multiple comparisons test |
| **2B**  **Frequency,**  **first**  **200 ms** | ArcLight  Bongwoori-R3  Bongwoori-Pos6 | < 0.0001 | **** | One-way ANOVA |
|  | ArcLight  Bongwoori-R3 | 0.7754 | ns | Tukey’s multiple comparisons test |
|  | ArcLight  Bongwoori-Pos6 | < 0.0001 | **** | Tukey’s multiple comparisons test |
|  | Bongwoori-R3  Bongwoori-Pos6 | < 0.0001 | **** | Tukey’s multiple comparisons test |
| **2B**  **Number of**  **cycles,**  **first**  **200 ms** | ArcLight  Bongwoori-R3  Bongwoori-Pos6 | < 0.0001 | **** | One-way ANOVA |
|  | ArcLight  Bongwoori-R3 | 0.3980 | ns | Tukey’s multiple comparisons test |
|  | ArcLight  Bongwoori-Pos6 | 0.0004 | *** | Tukey’s multiple comparisons test |
|  | Bongwoori-R3  Bongwoori-Pos6 | < 0.0001 | **** | Tukey’s multiple comparisons test |
| **2C**  **Peak of an oscillation** | ArcLight  Bongwoori-R3  Bongwoori-Pos6 | 0.0087 | ** | One-way ANOVA |
|  | ArcLight  Bongwoori-R3 | 0.0061 | ** | Tukey’s multiple comparisons test |
|  | ArcLight  Bongwoori-Pos6 | 0.1976 | ns | Tukey’s multiple comparisons test |
|  | Bongwoori-R3  Bongwoori-Pos6 | 0.3340 | ns | Tukey’s multiple comparisons test |
| **3C**  **Duration** | hSyn-ArcLight  CaMKIIα-ArcLight (short)  CaMKIIα-ArcLight (long)  PV-ArcLight | < 0.0001 | **** | One-way ANOVA |
|  | hSyn-ArcLight  CaMKIIα-ArcLight (short) | < 0.0001 | **** | Tukey’s multiple comparisons test |
|  | hSyn-ArcLight  CaMKIIα-ArcLight (long) | 0.8928 | ns | Tukey’s multiple comparisons test |
|  | hSyn-ArcLight  PV-ArcLight | > 0.9999 | ns | Tukey’s multiple comparisons test |
|  | CaMKIIα-ArcLight (short)  CaMKIIα-ArcLight (long) | < 0.0001 | **** | Tukey’s multiple comparisons test |
|  | CaMKIIα-ArcLight (short)  PV-ArcLight | < 0.0001 | **** | Tukey’s multiple comparisons test |
|  | CaMKIIα-ArcLight (long)  PV-ArcLight | 0.9353 | ns | Tukey’s multiple comparisons test |
| **3C**  **Frequency** | hSyn-ArcLight  CaMKIIα-ArcLight (short)  CaMKIIα-ArcLight (long)  PV-ArcLight | < 0.0001 | **** | One-way ANOVA |
|  | hSyn-ArcLight  CaMKIIα-ArcLight (short) | 0.0067 | ** | Tukey’s multiple comparisons test |
|  | hSyn-ArcLight  CaMKIIα-ArcLight (long) | 0.0595 | ns | Tukey’s multiple comparisons test |
|  | hSyn-ArcLight  PV-ArcLight | < 0.0001 | **** | Tukey’s multiple comparisons test |
|  | CaMKIIα-ArcLight (short)  CaMKIIα-ArcLight (long) | 0.6167 | ns | Tukey’s multiple comparisons test |
|  | CaMKIIα-ArcLight (short)  PV-ArcLight | 0.1341 | ns | Tukey’s multiple comparisons test |
|  | CaMKIIα-ArcLight (long)  PV-ArcLight | 0.0002 | *** | Tukey’s multiple comparisons test |
| **3C**  **Number of**  **cycles** | hSyn-ArcLight  CaMKIIα-ArcLight (short)  CaMKIIα-ArcLight (long)  PV-ArcLight | < 0.0001 | **** | One-way ANOVA |
|  | hSyn-ArcLight  CaMKIIα-ArcLight (short) | < 0.0001 | **** | Tukey’s multiple comparisons test |
|  | hSyn-ArcLight  CaMKIIα-ArcLight (long) | 0.9857 | ns | Tukey’s multiple comparisons test |
|  | hSyn-ArcLight  PV-ArcLight | 0.7274 | ns | Tukey’s multiple comparisons test |
|  | CaMKIIα-ArcLight (short)  CaMKIIα-ArcLight (long) | 0.0005 | *** | Tukey’s multiple comparisons test |
|  | CaMKIIα-ArcLight (short)  PV-ArcLight | < 0.0001 | **** | Tukey’s multiple comparisons test |
|  | CaMKIIα-ArcLight (long)  PV-ArcLight | 0.9466 | ns | Tukey’s multiple comparisons test |
| **3D**  **Peak of an oscillation** | hSyn-ArcLight  CaMKIIα-ArcLight (short)  CaMKIIα-ArcLight (long)  PV-ArcLight | 0.0104 | * | One-way ANOVA |
|  | hSyn-ArcLight  CaMKIIα-ArcLight (short) | 0.0443 | * | Tukey’s multiple comparisons test |
|  | hSyn-ArcLight  CaMKIIα-ArcLight (long) | 0.6060 | ns | Tukey’s multiple comparisons test |
|  | hSyn-ArcLight  PV-ArcLight | 0.4817 | ns | Tukey’s multiple comparisons test |
|  | CaMKIIα-ArcLight (short)  CaMKIIα-ArcLight (long) | 0.8741 | ns | Tukey’s multiple comparisons test |
|  | CaMKIIα-ArcLight (short)  PV-ArcLight | 0.0147 | * | Tukey’s multiple comparisons test |
|  | CaMKIIα-ArcLight (long)  PV-ArcLight | 0.1723 | ns | Tukey’s multiple comparisons test |
| **4B**  **Symmetry**  **Coefficient** | hSyn-ArcLight  CaMKIIα-ArcLight (short)  CaMKIIα-ArcLight (long)  PV-ArcLight | < 0.0001 | **** | One-way ANOVA |
|  | hSyn-ArcLight  CaMKIIα-ArcLight (short) | < 0.0001 | **** | Tukey’s multiple comparisons test |
|  | hSyn-ArcLight  CaMKIIα-ArcLight (long) | 0.6438 | ns | Tukey’s multiple comparisons test |
|  | hSyn-ArcLight  PV-ArcLight | 0.0984 | ns | Tukey’s multiple comparisons test |
|  | CaMKIIα-ArcLight (short)  CaMKIIα-ArcLight (long) | < 0.0001 | **** | Tukey’s multiple comparisons test |
|  | CaMKIIα-ArcLight (short)  PV-ArcLight | < 0.0001 | **** | Tukey’s multiple comparisons test |
|  | CaMKIIα-ArcLight (long)  PV-ArcLight | 0.7682 | ns | Tukey’s multiple comparisons test |
| **S3**  **Pearson**  **Correlation**  **Coefficient** | Filtered LFP  A single pixel at the tip | < 0.0001 | **** | Student’s *t*-test |
|  | Filtered LFP  1 pixel away from the tip | 0.0036 | ** | Student’s *t*-test |
|  | Filtered LFP  2 pixels away from the tip | 0.2313 | ns | Student’s *t*-test |
|  | Filtered LFP  4 pixels away from the tip | 0.0432 | * | Student’s *t*-test |
|  | Filtered LFP  7 pixels away from the tip | 0.0337 | * | Student’s *t*-test |

**^†^**Significance: * *p* < 0.05, ** *p* < 0.01, *** *p* < 0.001, **** *p* < 0.0001

**Supplementary Figure Legends**

**Fig S1.** Representative oscillation waveform of all oscillations analyzed in this article.

6 ArcLight, 6 Bongwoori-R3, and 5 Bongwoori-Pos6 oscillations are labeled and identified so that their representative oscillation waveforms may be compared to their movies in Movies S1, S2, and S3.

**Fig S2.** Examples of representative oscillation waveforms and their simultaneously acquired unfiltered and filtered local field potential (LFP) recordings.

Simultaneously acquired LFP recordings are 5-50 Hz bandpass filtered using the 5th order Butterworth high-pass and low-pass filters. For the oscillation in the top left and the oscillation in Fig 1D, filtered LFP recordings are noticeable and detectable. For the oscillations in the bottom left and right, filtered LFP recordings are neither noticeable nor detectable. GEVIs used in each of the 4 oscillations with their LFP recordings are as follows. Fig 1D: ArcLight, Top left: ArcLight, Bottom left: Bongwoori-R3, Bottom right: ArcLight.

**Fig S3.** Distance dependent dissociation of the LFP recording with the GEVI imaging.

The entire analysis was performed on the representative oscillation shown in Figure 1. On the left are location of pixels sampled, colored in yellow and red, to produce the optical activity waveforms shown in the middle. The white dotted lines indicate the location of the LFP recording glass micropipette. The top row is identical to Figure 1C and reproduced for reference. The second row shows the filtered LFP recording. The third to seventh rows show sampling the pixels progressively further away from the tip of the glass micropipette. The number of pixels sampled are as follows: third row = 1 pixel, fourth row = 8 pixels, fifth row = 40 pixels, sixth row = 100 pixels, seventh row = 154 pixels. The Pearson correlation coefficients between each optical recording and the LFP recording are calculated within the oscillation time window marked by the grey and red shaded boxes.

**Fig S4.** Representative k-nearest neighbor algorithm results for the three GEVIs.

For each recording, a k-nearest neighbor (KNN) algorithm trained on the first 500 ms of the baseline was used to score the rest of the recording. Distance on the left Y axis indicates how anomalous a 300 ms segment is from the 500 ms of the baseline. Larger distance values indicate the segment is more anomalous from the baseline. Distance values for the first 500 ms is 0 because the first 500 ms of the baseline was used to train each KNN algorithm. X axis indicates the frame number corresponding to the beginning of a 300 ms moving window. Each recording is 10000 frames, 1 ms per frame. Transparent red boxes indicate the position of oscillations detected in the recordings.

**Table S1.** *P* values and statistical tests.

*P* values, significances, and the exact name of the statistical tests performed in this article are summarized in the order of the figures in which they appear. For significance, we used a commonly accepted notation: * *p* < 0.05, ** *p* < 0.01, *** *p* < 0.001, **** *p* < 0.0001
